# Context-dependent roles of the NLRP3 inflammasome in osteomyelitis and strategies for precision modulation

**DOI:** 10.3389/fimmu.2026.1920166

**Published:** 2026-09-07

**Authors:** Lidan Yang, Lin Zhang, Xuxu Yang, Yang Yu

**Affiliations:** Department of Orthopedics, Affiliated Hospital of Zunyi Medical University, Zunyi, China

**Keywords:** bone remodeling, NLRP3 inflammasome, osteoimmunology, osteomyelitis, pyroptosis, therapeutic targets

## Abstract

Osteomyelitis is an inflammatory bone disease caused by pathogen infection, characterized by persistent infection, bone destruction, and impaired repair. Its refractoriness stems from the interplay between pathogen persistence and dysregulated host immune responses within the unique bone microenvironment. As a critical sensor of innate immunity, the NLRP3 inflammasome integrates pathogen-associated molecular patterns (PAMPs) and damage-associated molecular patterns (DAMPs), playing a pivotal regulatory role in the initiation and chronicity of osteomyelitis through caspase-1 activation, maturation and release of IL-1β and IL-18, and Gasdermin D (GSDMD)-mediated pyroptosis. This review systematically summarizes the activation mechanisms of the NLRP3 inflammasome within the bone immune microenvironment of osteomyelitis and its functions across multiple cell lineages. The local osteomyelitis microenvironment provides sustained NLRP3 activation signals through pathogen components, host damage signals, and bone matrix degradation products. Various cells, including macrophages, contribute to inflammatory amplification, enhanced bone resorption, or impaired repair via NLRP3-related pathways, forming a multi-layered pathological network through intercellular crosstalk. Mechanistically, four key axes operate: the IL-1β/receptor activator of nuclear factor-κB ligand (RANKL)/osteoclast axis driving infectious bone resorption; the GSDMD/pyroptosis/DAMPs axis sustaining a self-amplifying inflammatory loop; the reactive oxygen species (ROS)/mitochondria/autophagy axis providing the metabolic basis for persistent NLRP3 activation; and the osteoblast inhibition axis explaining post-infection repair failure. Notably, NLRP3 exhibits context-dependent roles in osteomyelitis—it may participate in antimicrobial defense during early infection, while in the chronic phase it primarily drives bone destruction and inadequate repair. Furthermore, *Staphylococcus aureus* can evade host defenses by attenuating NLRP3-mediated responses. Therapeutically, direct NLRP3 small-molecule inhibitors, caspase-1 inhibitors, GSDMD/pyroptosis inhibitors, IL-1β/IL-1R blockade, and mesenchymal stem cell (MSC)-derived exosome therapy all show potential value. However, the risk of systemic immunosuppression necessitates combining these strategies with adequate debridement and pathogen control. Local drug delivery systems, which achieve effective concentrations at the lesion while reducing systemic exposure, represent a more translationally promising direction. We propose that future NLRP3-targeted therapy for osteomyelitis should shift from “non-specific anti-inflammation” to “bone immune reprogramming”—stratified intervention based on infection stage, cell type, and inflammasome activation level, integrating NLRP3 modulation with antibacterial, anti-biofilm, anti-pyroptotic, and osteogenic-promoting strategies. This paradigm shift aims to transition from simple infection control to the reconstruction of a reparative bone immune microenvironment.

## 1 Introduction

Osteomyelitis is a refractory disease characterized by bone tissue infection, persistent inflammation, and disordered bone remodeling. Its clinical course often presents as protracted, recurrent, and difficult to eradicate, particularly in cases associated with *Staphylococcus aureus* infection (1). Unlike general soft tissue infections, osteomyelitis occurs within the dense, relatively hypovascular bone tissue where immune cell infiltration is restricted. Pathogens can not only reside within the medullary cavity and cortical bone but also form long-term occult infectious foci using necrotic bone, abscesses, biofilms, and internal fixation materials (2, 3). Even after standard antimicrobial therapy and surgical debridement, patients with chronic osteomyelitis may still experience bone destruction, bone defects, infection recurrence, and functional impairment. This indicates that the pathological basis of osteomyelitis is not simply pathogen replication, but rather the combined effect of persistent pathogen presence and dysregulated bone immunity (4–6).

In recent years, advances in osteoimmunology have transformed our understanding of osteomyelitis pathogenesis. Bone tissue is no longer viewed as a passively damaged target organ, but rather as a dynamic immune microenvironment composed of immune cells, bone cell lineages, vascular endothelial cells, bone marrow stromal cells, and extracellular matrix. Under infectious stimulation, macrophages, neutrophils, osteoblasts, osteocytes, osteoclast precursors, and bone marrow mesenchymal stem cells (BMSCs) all participate in pathogen recognition, release of inflammatory mediators, regulation of cell death, osteoclast activation, and bone repair modulation (7). When infection persists, necrotic tissue cannot be cleared in a timely manner, or biofilms persist long-term, the initially protective inflammatory response transforms into damaging mechanisms. These include sustained elevation of pro-inflammatory cytokines, excessive osteoclast activation, suppressed osteoblast function, impaired angiogenesis, and destruction of bone tissue structure (8). Thus, the core pathological process of osteomyelitis is essentially infection-driven disruption of bone immune homeostasis.

Among the numerous innate immune signaling pathways, the NOD-like receptor family pyrin domain containing 3 (NLRP3) inflammasome has emerged as a major focus in osteomyelitis research due to its ability to integrate both PAMPs and DAMPs (9, 10). Upon activation, the NLRP3 inflammasome promotes caspase-1 cleavage and activation, which subsequently processes pro-IL-1β and pro-IL-18 into mature inflammatory cytokines while simultaneously cleaving GSDMD to generate the N-terminal pore-forming fragment GSDMD-N, inducing pyroptosis (11). This effector axis can enhance anti-infective immune responses by promoting neutrophil recruitment and pathogen clearance on one hand, but on the other hand, it can amplify inflammatory damage and promote bone resorption and tissue destruction under chronic infection conditions (12–14). However, recent studies suggest that *S. aureus* can inhibit or delay macrophage NLRP3 responses during early infection, and NLRP3 deficiency does not significantly alter bacterial burden or bone homeostasis in some osteomyelitis models. This indicates that NLRP3 plays distinctly context-dependent roles in osteomyelitis, with its specific effects depending on pathogen type, infection stage, cell type, local tissue environment, and host immune status (15).

Based on this background, this review systematically summarizes the role of the NLRP3 inflammasome in the pathogenesis and progression of osteomyelitis. We first outline the bone immunopathological basis of osteomyelitis and the basic activation mechanisms of the NLRP3 inflammasome. Next, we analyze the specific mechanisms by which NLRP3 participates in osteomyelitis across multiple cell types, including macrophages, neutrophils, osteoblasts, osteocytes, osteoclast precursors, BMSCs, and vascular endothelial cells. We then delineate how four mechanistic pathways—the IL-1β/RANKL/osteoclast axis, the GSDMD/pyroptosis/DAMPs axis, the ROS/mitochondria/autophagy axis, and the osteoblast inhibition axis—mutually amplify each other to form a pathological network. Finally, we summarize the potential and limitations of NLRP3-targeted therapeutic approaches and drug development directions, and discuss the application prospects of NLRP3 inhibitors, IL-1β blockade, pyroptosis inhibition, exosome therapy, and local drug delivery systems in osteomyelitis treatment, aiming to provide new insights and a theoretical basis for precision therapy of osteomyelitis.

## 2 Pathological basis of osteomyelitis

### 2.1 The core challenge of osteomyelitis refractoriness lies in the combination of infection and bone structural peculiarities

Bone tissue possesses unique anatomical and physiological characteristics. Compared to soft tissues with rich blood supply and relatively open interstitial spaces, bone tissue is structurally dense, with limited blood supply within cortical bone, intricate canalicular networks, and a medullary cavity containing numerous hematopoietic cells, immune cells, adipocytes, and mesenchymal cells. Once infection enters bone tissue, pathogens can not only exist within the medullary cavity but also adhere to necrotic bone, bone matrix, internal fixation materials, or prosthetic surfaces, and even invade osteoblasts, osteocyte-like structures, or the canalicular network (16). Under these conditions, both antibiotics and immune cells struggle to achieve stable, adequate, and sustained effective concentrations locally, providing pathogens with a spatial basis for long-term persistence (17).

Taking *S. aureus* osteomyelitis as an example, this bacterium is one of the most common and representative pathogens in clinical osteomyelitis. Its pathogenicity stems not only from rapid proliferation and toxin production but also from its remarkable adaptability to the bone tissue environment. On one hand, *S. aureus* expresses multiple adhesion molecules that bind to bone matrix components such as collagen, fibronectin, and osteopontin, enabling firm adherence to bone surfaces. On the other hand, it can invade osteoblasts and establish a relatively occult intracellular survival state, evading antibodies, complement, and some antimicrobial agents (18, 19). In addition, biofilms can be formed by *Staphylococcus aureus* on internal fixation materials, necrotic bone, and bone surfaces. Bacteria within biofilms exhibit metabolic heterogeneity, with some subpopulations proliferating slowly, showing reduced antibiotic susceptibility, and resisting effective phagocytosis and killing by immune cells (8, 20). Therefore, even after prolonged antimicrobial therapy, infection recrudescence and chronic persistence may still occur. This characteristic indicates that the persistence of osteomyelitis is not merely a problem of “insufficient antibiotic potency, “ but rather that pathogens exploit the structural and microenvironmental features of bone tissue to form relatively closed, hypoperfused, and immunologically inaccessible ecological niches. Necrotic bone holds particular significance in this context. Lacking effective blood supply, necrotic bone provides an attachment surface for pathogens while simultaneously impeding antibiotic penetration (21). Persistent inflammatory cell infiltration, abscess formation, and tissue hypoxia surrounding necrotic bone further induce new bone cell death and bone matrix destruction (20). This establishes a vicious cycle of “infection—ischemia—necrosis—biofilm—re-infection”.

### 2.2 Host immune responses in osteomyelitis possess dual protective and damaging properties

Following infection, the host immune system senses PAMPs and DAMPs released from tissue injury through pattern recognition receptors (PRRs) expressed by neutrophils, macrophages, dendritic cells, and bone-resident cells such as osteoblasts and osteocytes (7). In early infection, these immune responses are critical for limiting bacterial spread. Neutrophils are rapidly recruited to the infection site, killing bacteria through phagocytosis, degranulation, ROS production, and neutrophil extracellular trap (NET) formation. Macrophages phagocytose bacteria and necrotic debris, produce inflammatory mediators, present antigens, and coordinate subsequent innate and adaptive immune responses. Thus, acute-phase inflammation initially serves predominantly protective functions (22). When an infectious focus persists, the role of neutrophils exhibits marked context dependence. NETs can capture extracellular bacteria and concentrate antibacterial proteins locally, limiting early bacterial dissemination. However, sustained or excessive NETosis also exposes histones and releases neutrophil elastase, cathepsin G, myeloperoxidase, matrix-degrading enzymes, and oxides. These mediators can damage osteoblast lineages and surrounding extracellular matrix, while NET components that are not cleared in a timely manner can act as DAMPs, continuously activating innate immune responses (23). Proteases and tissue damage products released during this process may further enhance RANKL-related osteoclast differentiation and bone resorption (24). Therefore, NETosis may help limit pathogens in early infection, but when it persists in the chronic phase, it may promote matrix damage and osteoclastic inflammation. Macrophage responses are similarly heterogeneous and cannot be simplistically categorized as a uniform “pro-inflammatory state.” Although the actual functional states of macrophages exist along a continuum, the M1/M2 framework remains useful for illustrating the imbalance between inflammatory and reparative programs (25). M1-like macrophages participate in antimicrobial defense through phagocytosis, ROS and reactive nitrogen species production, and release of inflammatory mediators such as IL-1β, tumor necrosis factor-α (TNF-α), and IL-6. However, when M1-like states predominate in persistent infection, they sustain local inflammatory signals, promote RANKL-dependent osteoclastogenesis, and expose osteoblasts, osteocytes, and mesenchymal stromal cells to a sustained inflammatory environment unfavorable for cell survival and osteogenic differentiation (26). In contrast, M2-like or inflammation-resolving macrophages promote apoptotic cell clearance, angiogenesis, matrix remodeling, and tissue repair (27). If macrophages fail to transition from a sustained inflammatory state to a reparative state in a timely manner, both enhanced bone resorption and suppressed new bone formation may occur concurrently. It should be noted that macrophage polarization in osteomyelitis is dynamic and spatially heterogeneous, with M1- and M2-associated features potentially coexisting within the same lesion; thus, it should not be understood as an absolute binary state. Adaptive immunity also participates in this balance between protection and damage. Inflammasome-derived IL-1β and other inflammatory cytokines promote the expansion or activation of IL-17-producing T cells, including Th17 and γδ T cells (28). IL-17 not only promotes neutrophil recruitment but also stimulates stromal cells, osteoblasts, and osteocytes to increase inflammatory mediator and RANKL expression, thereby indirectly linking adaptive immunity to osteoclast activation (29). Activated T cells themselves may also directly express RANKL. Regulatory T cells (Tregs) typically restrain excessive effector T cell and myeloid cell activation, but when Treg numbers are insufficient or their immunosuppressive function is impaired, sustained Th17/IL-17 responses cannot be effectively counterbalanced (30). The resulting Th17/Treg imbalance may promote chronic inflammation and infection-induced bone loss. However, direct evidence regarding how NLRP3 regulates distinct T cell subsets in osteomyelitis remains limited, with some mechanisms primarily derived from other infectious or inflammatory bone disease models. Therefore, the persistence of osteomyelitis cannot be simply attributed to overall immune insufficiency or overall immune hyperreactivity, but rather manifests as a functional mismatch: bacterial killing is insufficient, yet inflammatory tissue damage persists. *S. aureus* can interfere with phagosome maturation, phagosome-lysosome fusion, autophagy or xenophagy clearance, and intracellular antimicrobial mechanisms to survive within macrophages and bone-resident cells. Biofilm formation and slow-growth phenotypes further reduce bacterial susceptibility to host immunity and antibiotics. Consequently, robust inflammatory responses do not necessarily translate into effective pathogen clearance.

The NLRP3–caspase-1–GSDMD axis serves as a key mechanistic bridge linking “insufficient bacterial killing” and “excessive inflammation.” NLRP3 activation promotes caspase-1-dependent maturation of IL-1β and IL-18, and cleavage of GSDMD (14). The generated GSDMD-N fragment forms pores in the cell membrane, inducing pyroptosis in infected or stressed macrophages (31). As reviewed by Broz, Pyroptotic cells release mature inflammatory cytokines along with ATP, high-mobility group box 1 (HMGB1), mitochondrial components, and other DAMPs. These substances further recruit and activate immune cells and generate new NLRP3-activating signals (32). When pyroptosis can destroy intracellular bacterial niches and promote pathogen clearance, this response may be protective. However, when *S. aureus* is not cleared following inflammasome activation, repeated GSDMD-mediated membrane damage and DAMP release may continuously drive inflammasome activation, neutrophil recruitment, Th17-related inflammation, osteoclastogenesis, and bone matrix destruction, forming a self-amplifying injury loop. Conversely, insufficient or delayed NLRP3 responses may allow infected macrophages or bone-resident cells to remain viable, serving as intracellular bacterial reservoirs (33). Therefore, both insufficient and excessive inflammasome responses can produce detrimental outcomes under different conditions. This context-dependent model can explain two seemingly contradictory phenomena in chronic osteomyelitis: impaired pathogen clearance coexisting with persistent inflammatory tissue destruction. NLRP3 should thus not be viewed as a unidirectional switch that is always protective or always pathogenic, but rather as an immune regulatory node determined by infection stage, bacterial localization, responding cell types, and the balance between pathogen clearance and collateral tissue damage.

### 2.3 Bone remodeling imbalance in osteomyelitis manifests as concurrent enhanced resorption and suppressed formation

Bone destruction in osteomyelitis does not occur simply through direct bacterial “erosion” of bone tissue, but primarily through infection-induced bone remodeling imbalance. Normal bone remodeling depends on the balance between osteoblasts and osteoclasts: osteoclasts resorb old bone while osteoblasts form new bone, maintaining dynamic coordination. In osteomyelitis, this balance is disrupted by the inflammatory environment. On one hand, IL-1β, TNF-α, IL-6, prostaglandin E2 (PGE2), and RANKL are continuously elevated, promoting osteoclast generation and bone resorption. On the other hand, osteoblast function is impaired, with decreased osteogenic differentiation, collagen synthesis, and mineralization capacity, resulting in insufficient bone repair (34–36). Thus, a pathological state of “enhanced resorption with deficient formation” emerges locally. This process exhibits marked temporal and spatial heterogeneity. In the early acute phase of osteomyelitis, extensive inflammatory cell infiltration, local bone marrow edema, and abscess formation are observed within the medullary cavity. As the disease progresses, cortical bone destruction, periosteal reaction, sequestrum formation, and new bone encasement may coexist. That is, osteomyelitis is not a unidirectional bone loss process, but a complex remodeling process involving bone destruction, necrosis, reparative bone formation, and bone sclerosis simultaneously. Correspondingly, clinical imaging often reveals the coexistence of bone destruction and periosteal new bone formation, and pathological sections concurrently show inflammatory cell infiltration, necrotic bone, increased osteoclasts, and reparative bone formation. The NLRP3 inflammasome is highly relevant to this bone remodeling imbalance (37–41). Its downstream IL-1β promotes RANKL expression and osteoclast differentiation; GSDMD-mediated pyroptosis increases DAMP release, reinforcing inflammatory stimulation; and sustained inflammasome activation can suppress the osteogenic potential of osteoblasts and MSCs. Therefore, NLRP3 is not merely an inflammatory signaling pathway, but a crossroad node simultaneously affecting bone resorption, bone formation, and inflammation persistence.

## 3 Activation mechanisms of the NLRP3 inflammasome and its adaptive features in the osteomyelitis microenvironment

### 3.1 Basic composition and effector axis of the NLRP3 inflammasome

The canonical NLRP3 inflammasome is mainly composed of three components: the NLRP3 sensor, the ASC adaptor protein, and pro-caspase-1. The NLRP3 protein itself includes an N-terminal pyrin domain (PYD), a central NACHT domain, and a C-terminal leucine-rich repeat (LRR) region. The PYD is responsible for interaction with ASC; the NACHT domain is involved in ATP binding, hydrolysis, and oligomerization, serving as an important structural basis for NLRP3 conformational changes and inflammasome assembly; and the LRR region is associated with auto-inhibition and stimulus sensing (13, 42).

**Figure 1:**
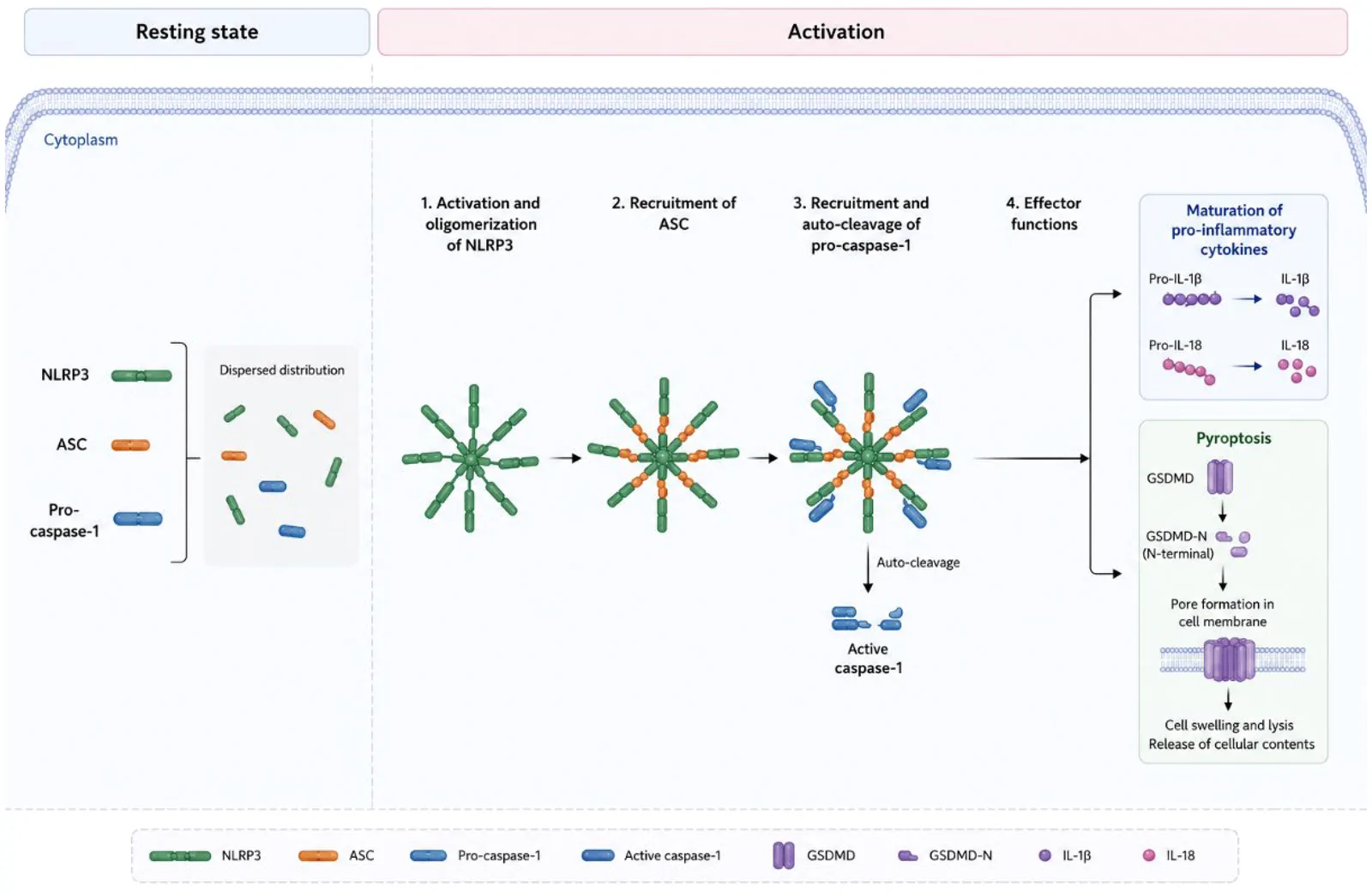
Basic composition and effector axis of the NLRP3 inflammasome. The canonical NLRP3 inflammasome comprises the sensor NLRP3, the adaptor ASC, and pro-caspase-1. Upon activation, NLRP3 oligomerizes and recruits ASC, which in turn recruits pro-caspase-1, leading to autocleavage and generation of active caspase-1. Active caspase-1 then cleaves pro-IL-1β and pro-IL-18 into their mature forms, and also cleaves GSDMD to release the pore-forming GSDMD-N. GSDMD-N inserts into the plasma membrane, forming pores that cause pyroptosis and release of intracellular contents, including mature IL-1β, IL-18, and DAMPs. These effector molecules amplify local inflammation and modulate immune responses, linking pathogen recognition to inflammatory bone destruction.

In the resting state, NLRP3 exists in a relatively inhibited conformation. When cells are stimulated by infection, injury, or metabolic stress, NLRP3 undergoes conformational changes and oligomerizes, subsequently recruiting ASC. ASC further recruits pro-caspase-1 through its caspase recruitment domain (CARD), bringing multiple pro-caspase-1 molecules into proximity to undergo autocleavage, generating active caspase-1. Active caspase-1 has two key functions: first, it processes pro-IL-1β and pro-IL-18 into mature IL-1β and IL-18; second, it cleaves GSDMD to generate the pore-forming GSDMD-N fragment. The GSDMD-N fragment inserts into the cell membrane to form pores, leading to leakage of cellular contents, cell swelling, and pyroptosis (43, 44). This effector axis possesses strong inflammatory amplification capacity. IL-1β induces local cells to release additional chemokines and inflammatory factors, promoting neutrophil and monocyte recruitment. IL-18 regulates natural killer (NK) cell and T cell responses, enhancing interferon-γ (IFN-γ)-related immune effects. Pyroptosis further activates surrounding cells by releasing cellular contents, DAMPs, and mature inflammatory factors (45). For acute infection, this response helps rapidly mobilize the immune system. However, for chronic, focal, and difficult-to-eradicate infections like osteomyelitis, sustained NLRP3 activation may lead to uncontrolled inflammation and bone tissue damage (46) (**Figure 1**).

### 3.2 The “two-step model” of NLRP3 activation and its manifestation in osteomyelitis

NLRP3 inflammasome activation can generally be divided into a priming step and an activation step. The priming step is mainly mediated by receptors such as Toll-like receptors (TLRs), TNF receptor (TNFR), and IL-1R, which upregulate NLRP3 and pro-IL-1β expression through nuclear factor-κB (NF-κB) signaling. In the absence of priming signals, intracellular NLRP3 and pro-IL-1β levels are low, and even upon receiving activating stimuli, sufficient IL-1β release is difficult to achieve. The activation step is triggered by signals of cellular homeostasis disruption, including K^+^ efflux, aberrant Ca²^+^ flux, mitochondrial damage, increased ROS, ATP release, lysosomal rupture, and membrane pore formation (45). The osteomyelitis microenvironment almost simultaneously provides the stimulatory conditions required for both steps. Pathogen components such as *S. aureus* lipoteichoic acid, peptidoglycan, lipoproteins, and toxins activate NF-κB through TLR2, providing priming signals for NLRP3 expression and pro-IL-1β synthesis (47). Meanwhile, local cell necrosis, abscess formation, tissue hypoxia, acidic environment, and oxidative stress induce mitochondrial damage, ATP release, and ionic flux abnormalities, providing activation signals for NLRP3 assembly (45). The long-term presence of necrotic bone and biofilms ensures that these stimuli are not transient, but rather recurrent, low-grade, and persistent. Consequently, NLRP3 activation in osteomyelitis exhibits chronic and spatially heterogeneous characteristics.

NLRP3 activation depends not only on the intensity of priming and activating signals but is also finely regulated by multiple post-translational modifications that affect its protein stability, subcellular localization, conformational state, and ability to recruit NEK7 and ASC. Inhibitory ubiquitination can maintain NLRP3 in a low-activity state or promote its degradation, while priming- or activation-induced deubiquitination can relieve this inhibition and promote inflammasome assembly (48). Phosphorylation regulates NLRP3 in a site-specific manner: phosphorylation at certain sites helps maintain NLRP3 auto-inhibition, whereas phosphorylation or dephosphorylation at other sites can promote deubiquitination, self-aggregation, and downstream signaling. For example, JNK1-mediated phosphorylation of NLRP3 at Ser194 promotes NLRP3 deubiquitination and self-aggregation, serving as a critical event in inflammasome priming (49). Additionally, phosphorylation of mouse NLRP3 at Ser803 in the LRR domain—corresponding to human NLRP3 Ser806—during the priming step restricts NEK7 recruitment; dephosphorylation of this site during the activation step facilitates NEK7 and BRCC3 recruitment, NLRP3 deubiquitination, and inflammasome assembly (50). A recently identified CK2–PGK1–NLRP3 regulatory axis shows that CK2-mediated phosphorylation of PGK1 at Ser271 activates its non-glycolytic protein kinase function, and PGK1 subsequently phosphorylates NLRP3 at Ser448/Ser449, promoting USP14 recruitment and NLRP3 deubiquitination, thereby enhancing inflammasome activation (51). Although the roles of these specific sites and regulatory axes in osteomyelitis lack direct experimental validation, these mechanisms provide a molecular basis for explaining how bacterial virulence factors, oxidative stress, or therapeutic agents modulate inflammasome activity without significantly altering total NLRP3 expression. Immunometabolic status represents another important checkpoint. Increased glycolytic flux supports NF-κB-dependent priming, pro-IL-1β synthesis, ATP generation, and pro-inflammatory macrophage function. Mitochondrial dysfunction generates mitochondrial ROS (mtROS) and oxidized mitochondrial DNA (mtDNA), which, upon entering the cytosol, can bind to the NLRP3 inflammasome complex and further enhance NLRP3 activation (52). Glycolysis can also regulate NLRP3 through metabolite-dependent post-translational modifications. Recent studies show that glycolysis-derived lactate induces AARS2-mediated NLRP3 lactylation, with lactylation at Lys24 and Lys565 promoting ASC recruitment and inflammasome assembly (53). In contrast, fatty acid oxidation and oxidative phosphorylation are generally associated with macrophage survival, inflammation resolution, and tissue repair programs, though their specific effects are highly dependent on cell type, tissue microenvironment, and infection stage. In osteomyelitis, hypoxia, nutrient competition, bacterial metabolism, and local hypoperfusion may drive immune cells and bone-resident cells to switch between glycolytic inflammatory states and oxidative metabolism- or lipid-dependent persistent infection states. Future metabolic flux studies in infected bone tissue and cell-specific models are needed to clarify whether these metabolic programs promote bacterial killing, inflammasome activation, immune tolerance, or bone tissue repair.

In osteomyelitis caused by Gram-negative or polymicrobial infections, the non-canonical inflammasome pathway should also be considered. Cytosolic lipopolysaccharide (LPS) can directly bind and activate human caspase-4 and caspase-5 or mouse caspase-11, inducing their oligomerization and activation (54). Activated caspase-4/5/11 can directly cleave GSDMD in a caspase-1-independent manner, releasing the pore-forming GSDMD-N fragment, which increases membrane permeability, induces pyroptosis, and promotes K^+^ efflux (55). The resulting ionic homeostasis disruption can secondarily activate the canonical NLRP3–ASC–caspase-1 pathway, promoting effective maturation and release of IL-1β and IL-18 (56). Therefore, the non-canonical inflammasome can function both as a direct pyroptosis-inducing effector pathway and as an upstream amplifier of the NLRP3 inflammasome. However, the specific contribution of this pathway to Gram-negative, diabetic foot-related, post-traumatic, or implant-associated osteomyelitis remains systematically understudied. Notably, the inflammasome biology revealed by existing studies is not limited to the single NLRP3–caspase-1 pathway. Studies in other infectious, inflammatory, or tissue injury diseases suggest that NAIP–NLRC4, AIM2, non-canonical inflammasomes, and PANoptosis may jointly participate in pathogen recognition, inflammatory mediator release, and inflammatory cell death. as reviewed by Monack, The NAIP–NLRC4 inflammasome primarily responds to cytosolic bacterial flagellin and needle or rod proteins of the type III secretion system, with NAIP proteins responsible for ligand recognition and NLRC4 participating in inflammasome assembly and caspase-1 activation (57). AIM2 recognizes aberrantly localized cytosolic double-stranded DNA, including bacterial DNA released during infection and host-derived DNA from damaged cells or organelles; in some tissue injury models, mtDNA may also participate in AIM2 inflammasome activation (58). Although these mechanisms are primarily derived from other infection or tissue injury models, they possess biological plausibility in osteomyelitis as well: osteomyelitis lesions may simultaneously contain intracellular pathogen components, bacterial nucleic acids, necrotic bone, and DAMPs released from damaged cells, especially in Gram-negative, mixed, and implant-associated infections, potentially creating conditions for parallel or sequential activation of multiple inflammasomes. However, direct evidence for the specific roles and relative contributions of NAIP–NLRC4, AIM2, or PANoptosis in osteomyelitis is currently lacking. Future studies should employ cell-specific approaches and multi-pathway marker detection across different pathogens and clinical subtypes to determine whether these inflammasomes and cell death programs participate in antimicrobial defense, bone destruction, and repair impairment in osteomyelitis.

### 3.3 Major sources of NLRP3-activating signals in osteomyelitis

**Figure 2:**
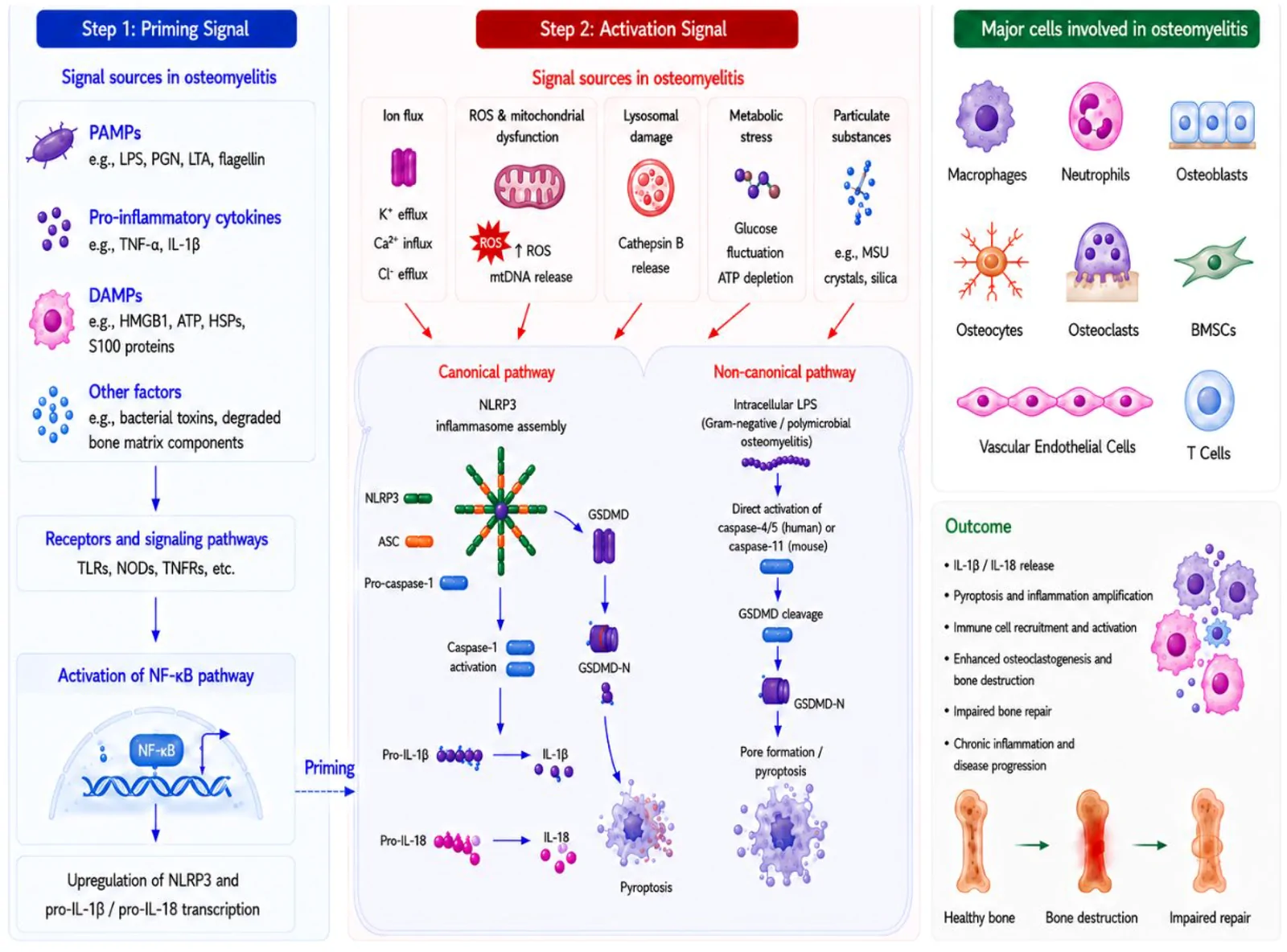
Canonical and non-canonical inflammasome activation pathways in the osteomyelitis microenvironment. This image systematically illustrates the integrated activation mechanisms of the canonical and non-canonical NLRP3 inflammasome pathways within the osteomyelitis microenvironment. The canonical NLRP3 inflammasome pathway strictly follows a two-signal priming-activation model. During the priming phase, PAMPs, pro-inflammatory cytokines, DAMPs and other factors activate the NF-κB pathway through TLRs, NOD-like receptors, and TNFRs. This upregulates the transcription of NLRP3 and pro-IL-1β/pro-IL-18, providing the molecular basis for inflammasome assembly. In the activation phase, multiple cellular homeostatic disruptions collectively trigger NLRP3 oligomerization and its assembly with ASC and pro-caspase-1 into the active inflammasome complex, leading to caspase-1 activation. Active caspase-1 subsequently cleaves pro-IL-1β and pro-IL-18 into their mature forms, while also cleaving GSDMD to generate the GSDMD-N fragment, which forms membrane pores, induces pyroptosis, and promotes the release of IL-1β, IL-18, and intracellular DAMPs. Concurrently, in Gram-negative or polymicrobial osteomyelitis, intracellular LPS directly binds and activates human caspase-4/5 or mouse caspase-11. These activated caspases can directly cleave GSDMD independent of caspase-1, triggering pore formation and pyroptosis. The major cell types involved in this process—including macrophages, neutrophils, osteoblasts, osteocytes, osteoclasts, bone marrow mesenchymal stem cells, and T cells—participate in or are affected by inflammasome activation in a cell-type- and context-dependent manner, collectively forming the pathological network of osteomyelitis. The integrated activation of both pathways eventually leads to a series of pathological outcomes: release of mature IL-1β and IL-18, pyroptosis and inflammatory amplification, recruitment and activation of immune cells, enhanced osteoclastogenesis and bone destruction, impaired bone repair, and sustained chronic inflammation, driving the transition from healthy bone to progressive bone destruction and defective regeneration in osteomyelitis.

NLRP3-activating signals in osteomyelitis can be roughly divided into three categories (45, 59, 60). The first category comprises pathogen-derived signals. Cell wall components and secreted toxins of *S. aureus* can directly or indirectly induce NLRP3 inflammasome activation. Bacterial toxins cause cell membrane damage, leading to K^+^ efflux, which is one of the important common mechanisms of NLRP3 activation. Bacterial infection can also cause lysosomal stress, mitochondrial dysfunction, and ROS generation, further enhancing inflammasome responses (61, 62). It is noteworthy that different pathogens have distinct effects on NLRP3. Some bacteria strongly induce inflammasome activation, while *S. aureus* may exhibit complex effects of both induction and inhibition at different infection stages. Recent studies suggest that after *S. aureus* infection of macrophages, the NLRP3 inflammasome response is not consistently robust and may even be suppressed or delayed at certain stages (63). This finding indicates that NLRP3 activation in osteomyelitis cannot be simplistically interpreted as “more bacteria, stronger NLRP3, “ but rather should be analyzed in conjunction with pathogen immune evasion strategies. The second category comprises host damage-derived signals. Extensive cell death, necrotic bone formation, and tissue hypoxia in osteomyelitis release ATP, HMGB1, mtDNA, uric acid-like substances, and other DAMPs. These substances activate NLRP3 through pathways such as the P2X7 receptor, ROS generation, mitochondrial damage, and lysosomal stress. Compared to pathogen components, damage-associated signals better explain the persistence of inflammation in chronic osteomyelitis. Even when bacterial burden temporarily decreases, necrotic bone and dead cells continue releasing danger signals, keeping NLRP3 in a low-level activation state and impeding inflammation resolution. The third category comprises bone matrix-derived signals. Following bone tissue destruction, collagen fragments, mineralized matrix components, hydroxyapatite particles, and other bone matrix degradation products may participate in inflammatory responses as local danger signals. Although the specific mechanisms by which bone matrix components activate NLRP3 require further investigation, from the perspective of the osteomyelitis pathological environment, bone destruction itself may not only be a consequence but also a cause of persistent inflammation. Components released upon bone matrix destruction can further stimulate immune cells and osteoclast precursors, creating a positive feedback loop between bone resorption and inflammation (**Figure 2**).

## 4 Roles and mechanisms of NLRP3 inflammasome activation in different cell types in osteomyelitis

### 4.1 Macrophages

Macrophages are among the most important pathogen-recognizing cells in the early stage of osteomyelitis infection. After *S. aureus* enters bone tissue, macrophages sense bacterial cell wall components, lipoproteins, toxins, and extracellular nucleic acids through TLR2, TLR4, NOD-like receptors (NLRs), and other PRRs. TLR signaling activates NF-κB, upregulating NLRP3 and pro-IL-1β expression and providing priming conditions for inflammasome assembly. Subsequently, bacterial toxin-induced membrane damage, K^+^ efflux, ATP release, mitochondrial damage, and elevated ROS provide activating signals, enabling NLRP3 to assemble with ASC and pro-caspase-1 into the inflammasome. Activated caspase-1 promotes the maturation and release of IL-1β and IL-18, and induces pyroptosis through GSDMD cleavage (64–67). The classical view holds that this process facilitates early antimicrobial responses, as IL-1β promotes neutrophil recruitment, IL-18 enhances IFN-γ-related responses, and pyroptosis helps expose intracellular pathogens and trigger stronger inflammatory clearance mechanisms.

However, in osteomyelitis, macrophage NLRP3 activation does not always translate into effective antimicrobial activity. *S. aureus* possesses robust immune evasion capabilities, affecting macrophage function through intracellular survival, virulence factor regulation, biofilm formation, and modulation of host cell metabolic states. A recent study based on bone marrow-derived macrophages and experimental *S. aureus* osteomyelitis models provides an important revision: following *S. aureus* infection of macrophages, the NLRP3 inflammasome response is not rapid, robust, and sustained, but rather relatively weak and delayed. More notably, in experimental *S. aureus* osteomyelitis, NLRP3 deficiency did not significantly alter bacterial burden control or bone homeostasis (68). This suggests that NLRP3 in *S. aureus* osteomyelitis is not necessarily a core defense mechanism that effectively limits pathogens, but rather may be an immune node that pathogens evade or attenuate. This finding forms a valuable contrast with earlier studies emphasizing the pro-inflammatory role of NLRP3. Previous studies often highlighted that *S. aureus* activates NLRP3, promotes IL-1β release and pyroptosis, thereby exacerbating infectious inflammatory damage. For example, studies on *S. aureus* α-toxin showed that the toxin triggers NLRP3-dependent IL-1β secretion, indicating that bacterial virulence factors can indeed serve as important sources of NLRP3 activation (69). Recent evidence suggests that in bone marrow-derived macrophages, *S. aureus* does not simply activate macrophage NLRP3 signaling but can temporarily suppress or delay this response during early intracellular infection (70, 71). Specifically, ASC speck formation, caspase-1 activation, IL-1β release, GSDMD cleavage, and pyroptosis during the initial 18 hours of infection were all relatively limited, only gradually increasing at later time points. Furthermore, infected macrophages showed weaker responses to subsequent stimulation, suggesting that *S. aureus* may induce a state of reduced NLRP3 reactivity rather than simply failing to provide sufficient activation signals. Delayed inflammasome activation may reduce early pyroptotic clearance of infected cells, thereby maintaining an intracellular niche where bacteria can evade extracellular immune effects. However, it remains unclear which specific bacterial factors and host molecular checkpoints mediate this inhibition process.

Mechanistically, two distinct but coexisting pathways link macrophage NLRP3 to osteomyelitis chronicity. The first is the “hyperinflammation pathway”: infectious stimuli cause macrophages to continuously produce IL-1β and IL-18, and pyroptosis releases cellular contents, further activating surrounding immune cells and bone cells, leading to inflammatory amplification and bone destruction. The second is the “defense failure pathway”: *S. aureus* survives within macrophages or modulates macrophage responses through virulence factors, preventing NLRP3 from adequately activating effective bactericidal programs, allowing pathogens to persist and inducing low-grade, recurrent, and chronic inflammation. These two pathways are not contradictory; together, they explain the coexistence of “insufficient pathogen clearance” and “excessive inflammatory damage” in chronic osteomyelitis. Therefore, therapeutic targeting of macrophage NLRP3 cannot be simplistically understood as inflammasome inhibition. If NLRP3 is excessively inhibited in early infection, IL-1β-mediated neutrophil recruitment and antimicrobial responses may be weakened. If, after infection has been controlled by antibiotics and debridement, local macrophages continue to undergo pyroptosis and release IL-1β, inhibiting NLRP3 may help reduce inflammatory bone destruction. Thus, macrophage NLRP3 represents the most controversial yet therapeutically valuable cellular node in osteomyelitis, its significance lying not in simply being “pro-inflammatory” or “anti-inflammatory, “ but in reflecting the dynamic balance between host defense and pathogen evasion.

### 4.2 Neutrophils

Neutrophils are among the earliest and most abundant effector cells in acute suppurative osteomyelitis. Their main functions include bacterial phagocytosis, ROS release, degranulation, production of proteolytic enzymes, and NET formation (72). In the acute phase of osteomyelitis, insufficient neutrophil recruitment leads to pathogen spread. However, persistent neutrophil infiltration can damage bone matrix, vascular endothelium, and osteoblasts through release of myeloperoxidase, elastase, matrix metalloproteinases, and ROS (73). IL-1β is an important factor driving neutrophil recruitment; therefore, NLRP3 activation in macrophages or local bone cell lineages can indirectly enhance neutrophil infiltration. However, whether neutrophils themselves primarily depend on NLRP3 for IL-1β production is inconsistent across studies. Studies on the interaction between human neutrophils and community-acquired methicillin-resistant *S. aureus* (MRSA) showed that neutrophils released IL-1β after phagocytosing live bacteria, but this process was independent of the NLRP3 inflammasome or caspase-1, instead depending on receptor-interacting protein kinase 3 (RIPK3) and serine proteases (74). In contrast, nigericin-stimulated human neutrophils exhibited NLRP3-related IL-1β production (75). These results indicate that IL-1β release mechanisms in neutrophils are stimulus-dependent: even for IL-1β elevation, different stimuli may engage NLRP3-dependent or NLRP3-independent pathways. This is important for osteomyelitis research. If only IL-1β elevation in infected bone tissue is measured, one cannot directly infer that it all originates from the NLRP3 inflammasome. In osteomyelitis lesions with extensive neutrophil infiltration, IL-1β may arise from macrophage NLRP3 activation, osteoblast inflammatory responses, or neutrophil RIPK3 and protease-related pathways (7, 15).

From a pathological perspective, neutrophils participate in osteomyelitis with two opposing effects. In the acute phase, neutrophils are indispensable for controlling suppurative infection. In the chronic phase, persistent neutrophil infiltration and repeated activation may exacerbate bone tissue damage. NLRP3 in this context primarily acts as an indirect modulator: through IL-1β production by macrophages, osteoblasts, or other cells, it influences neutrophil recruitment. However, neutrophils themselves can also release IL-1β and damaging mediators through NLRP3-independent mechanisms (76). Therefore, even if NLRP3-targeted therapy reduces some IL-1β, it may not completely block neutrophil-mediated inflammatory damage in osteomyelitis.

### 4.3 Osteoblasts

Osteoblasts are the core cells responsible for bone formation and repair. However, in osteomyelitis, they are also important participants in pathogen attack and immune responses. *S. aureus* can bind to osteoblast surfaces through adhesion molecules and invade the intracellular compartment. Intracellularly surviving bacteria are not easily cleared by antibodies, complement, and some antimicrobial agents, making osteoblasts a potential reservoir for occult pathogen persistence (77). Simultaneously, upon stimulation by bacterial components, toxins, IL-1β, TNF-α, and ROS, osteoblasts release IL-6, IL-8, monocyte chemoattractant protein-1 (MCP-1), RANKL, and other cytokines, promoting immune cell recruitment and osteoclastogenesis. Thus, osteoblasts are both damaged bone-forming cells and active signal cells within the infection-inflammation network.

The connection between the NLRP3 inflammasome and osteoblast dysfunction is mainly reflected in two aspects. First, NLRP3 activation enhances local inflammatory responses through IL-1β and IL-18 release, exposing osteoblasts to a sustained pro-inflammatory microenvironment. Second, the NLRP3–caspase-1–GSDMD axis can induce osteoblast pyroptosis, leading to cell membrane rupture, release of inflammatory contents, and decreased osteogenic function (78). Studies on osteoblast pyroptosis have shown that TNF-α stimulation of MC3T3-E1 osteoblastic precursor cells induces GSDMD/GSDME-mediated pyroptosis through an ELP2–STAT3–NLRP3-related mechanism, affecting osteogenic differentiation; ELP2 silencing inhibits the NLRP3–GSDMD/GSDME pathway and alleviates pyroptosis (79). Direct evidence from osteomyelitis patients and *S. aureus* mouse osteomyelitis models also supports the involvement of pyroptosis in infectious bone destruction. One study comparing infected bone fragments from osteomyelitis patients with non-infected bone tissue found significantly elevated NLRP3, caspase-1, and GSDMD expression in infected bone; in an *S. aureus*-induced mouse osteomyelitis model, pyroptosis-related proteins were similarly upregulated, and inhibition of pyroptosis-related proteins alleviated inflammatory responses and bone destruction (80). Although this study did not fully localize all pyroptotic signals specifically to osteoblasts, the overall enhancement of pyroptosis in infected bone fragments suggests that osteoblasts, osteocytes, and immune cells within bone tissue may all participate in this process.

From the perspective of pathological consequences, the harm of osteoblast NLRP3 activation is not merely “releasing more inflammatory factors, “ but rather causing “osteoblast dysfunction” in the bone remodeling imbalance of osteomyelitis (81). On one hand, osteoblast pyroptosis reduces the number of cells involved in bone formation. On the other hand, inflammatory stimuli suppress osteogenic differentiation programs such as Runt-related transcription factor 2 (RUNX2), alkaline phosphatase (ALP), and osteocalcin (OCN), reducing the mineralization capacity of surviving osteoblasts. Furthermore, osteoblast release of RANKL promotes osteoclast differentiation, further enhancing bone resorption (82). Thus, NLRP3-related responses in osteoblasts simultaneously produce both “insufficient repair” and “enhanced destruction.” Therefore, osteoblasts are a cell type that cannot be overlooked in NLRP3 osteomyelitis research, as they can actively participate in disease progression through inflammasome and pyroptosis pathways.

### 4.4 Osteocytes

Compared to osteoblasts, osteocytes are embedded deep within the mineralized bone matrix in the lacunar-canalicular network, possess long lifespans, and are relatively inaccessible to professional immune cells. Therefore, in osteomyelitis, they may serve as an important locus for maintaining long-term infection, persistent inflammation, and repair impairment (83). Following local hypoxia, acidification, ROS accumulation, and bacterial toxin exposure, osteocytes may develop cellular dysfunction and may further undergo apoptosis, necrosis, pyroptosis, or other forms of regulated cell death. More importantly, osteocytes may provide a particularly favorable niche for persistent intracellular *S. aureus* infection: osteocytes are long-lived, located deep within the lacunar-canalicular network where circulating immune cells cannot adequately access, and the intracellular bacterial location also reduces the likelihood of complete clearance by antimicrobial agents (84). In human osteocyte-like cells, intracellular *S. aureus* can establish stable long-term infection and gradually transition to a low-metabolic, slow-growing small colony variant (SCV)-like phenotype during persistent infection. This phenotypic transition may reduce bacterial antibiotic sensitivity and antigen exposure while retaining the potential for later reactivation (85). NLRP3-related research in osteocytes is currently less extensive than in macrophages and osteoblasts, but recent osteomyelitis studies have begun to incorporate osteocyte pyroptosis into disease mechanisms. A recent study showed that in *S. aureus* protein A-induced injury models of MC3T3-E1 osteoblasts and MLO-Y4 osteocytes, NLRP3-mediated pyroptosis and autophagy-related molecules were activated; FTO-engineered extracellular vesicles (EVs) could downregulate NLRP3 in an m6A demethylation-dependent manner, suppress autophagy and pyroptosis, and attenuate bone injury in a mouse osteomyelitis model (86). The importance of this study lies in extending NLRP3 research in osteomyelitis from traditional immune cells to osteocytes and bone-repairing cells, suggesting that osteocyte pyroptosis may be a significant cause of persistent infectious bone destruction.

The pathological impact of osteocyte NLRP3 activation can be understood at three levels. First, osteocyte death weakens homeostatic regulation within the bone matrix. Osteocytes communicate with osteoblasts and bone surface cells through the canalicular network; once extensive death occurs, the bone tissue’s responsiveness to mechanical stimuli and repair signals declines. Second, osteocyte pyroptosis releases IL-1β, IL-18, and DAMPs, forming a sustained inflammatory source deep within the bone matrix, so that inflammation is not confined to the medullary cavity surface. Third, osteocytes can regulate RANKL expression; if infection and inflammation bias osteocytes toward a pro-osteoclastogenic phenotype, osteoclast recruitment and bone resorption may be enhanced (87–89). Thus, osteocytes are not simply passive structural cells that die, but may serve as important carriers of “deep inflammatory memory” in chronic osteomyelitis.

From a therapeutic perspective, osteocyte NLRP3 holds special value. Macrophage NLRP3 inhibition may affect antimicrobial immunity, whereas osteocyte NLRP3 inhibition is more oriented toward tissue protection and bone repair. However, because osteocytes are located deep within the bone matrix, drug delivery is challenging, and systemic administration alone cannot precisely target osteocytes. A more feasible future direction involves bone-targeting nanoparticles, biodegradable bone-filling materials, multifunctional hydrogels, or EV-based drug delivery (90–93), enabling NLRP3-modulating molecules to reach bone injury regions, reduce osteocyte pyroptosis, and protect the bone matrix network.

### 4.5 Osteoclasts and their precursors

Osteoclasts are the direct executing cells of bone resorption, originating from the monocyte-macrophage lineage. Bone destruction in osteomyelitis must ultimately manifest through osteoclast activation and bone matrix resorption. Therefore, osteoclasts and their precursors are key cells connecting inflammatory responses to structural bone damage. Locally sustained elevation of IL-1β, TNF-α, IL-6, and RANKL jointly promotes the differentiation of osteoclast precursors into mature osteoclasts. NLRP3 in this process can both indirectly enhance osteoclastogenesis by promoting IL-1β release and directly participate in differentiation and activation programs within osteoclast precursors (94).

Studies on the relationship between NLRP3 and osteolysis provide important foundational evidence. Research on NLRP3 gain-of-function mutation-associated bone diseases showed that NLRP3 promotes osteolysis not only through inflammation-dependent mechanisms but also through inflammation-independent effects on osteoclasts and bone metabolism, indicating that the connection between NLRP3 and bone resorption is not merely an indirect IL-1β-mediated link (95, 96). Although these results derive from NLRP3-associated autoinflammatory bone diseases, they are instructive for osteomyelitis: once the infectious environment continuously activates NLRP3, the osteoclast system may be driven not only by inflammatory cytokines but also by NLRP3-related intracellular programs directly. Infectious osteoclast enhancement in osteomyelitis is also closely associated with pyroptosis. Studies showing elevated NLRP3, caspase-1, and GSDMD in *S. aureus* osteomyelitis models, with pyroptosis inhibition alleviating bone destruction, suggest that IL-1β and DAMPs released by pyroptosis promote an osteoclast-favorable microenvironment (80). In other words, osteoclast activation arises not only from direct RANKL stimulation but also from inflammatory contents released by pyroptotic cells. Osteoclast NLRP3 research requires special attention to one issue: osteoclasts and macrophages share a common origin. Therefore, many *in vitro* studies use RAW264.7 cells or bone marrow-derived monocytes as osteoclast precursors, but these cells can exhibit either macrophage inflammatory features or enter osteoclast differentiation programs under different induction conditions (97). Thus, whether observed NLRP3 upregulation primarily represents inflammasome activation or inflammatory-like reprogramming accompanying osteoclast differentiation needs to be interpreted in conjunction with markers such as tartrate-resistant acid phosphatase (TRAP) staining, resorption pit assays, nuclear factor of activated T cells c1 (NFATc1), cathepsin K, and matrix metalloproteinase 9 (MMP9).

From a pathological analysis perspective, the effect of NLRP3 on osteoclasts is a major reason for irreversible bone destruction in osteomyelitis. Antibiotics can reduce pathogen burden, but already activated osteoclast programs and inflammatory bone resorption may persist for a short period. This explains why some patients may still develop bone defects and structural instability even after infection is controlled. Therefore, the significance of NLRP3-targeted therapy at the osteoclast level is not to replace antimicrobial therapy, but to limit excessive bone resorption after pathogen control, creating conditions for subsequent osteogenic repair.

### 4.6 Bone marrow mesenchymal stem cells

Bone marrow mesenchymal stem cells (BMSCs) are an important source for bone repair and regeneration, capable of differentiating into osteoblasts, chondrocytes, and adipocytes, and modulating immune responses through paracrine effects. In chronic osteomyelitis, persistent infection, inflammatory cytokines, ROS, hypoxia, and cell death signals impair the proliferation, migration, and osteogenic differentiation of MSCs, hindering repair of infection-induced bone defects. A close connection exists between the NLRP3 inflammasome and MSC dysfunction: the inflammatory environment can activate NLRP3 in MSCs or their surrounding cells, increasing IL-1β and IL-18, which further suppresses osteogenic differentiation (98). NLRP3-related pyroptosis may also reduce the number of cells with reparative potential.

Unlike macrophages and osteoclasts, the research value of MSCs in osteomyelitis lies not only in whether they undergo NLRP3 activation, but also in their potential as therapeutic tools for NLRP3 modulation. MSC-derived exosomes contain miRNAs, proteins, and lipid molecules that can be taken up by macrophages, osteoblasts, osteocytes, and endothelial cells, thereby modulating inflammation, pyroptosis, autophagy, and osteogenesis. miR-223 is one of the miRNAs that has received considerable attention in NLRP3 modulation research. Although conclusions vary across different disease models, multiple studies support that MSCs or their exosomes can regulate NLRP3-related inflammation through miRNAs. For example, studies showing that MSCs attenuate tissue damage through the miR-223/NLRP3 pathway demonstrate a clear connection between miR-223 and NLRP3 regulation (99). For osteomyelitis, miR-223 or other NLRP3-negative-regulating miRNAs hold potential value: they may reduce excessive macrophage inflammation, decrease osteoblast and osteocyte pyroptosis, and improve the osteogenic microenvironment of MSCs themselves (100).

However, MSC exosome therapy also requires careful consideration. Osteomyelitis is an active infectious disease, and excessive immunosuppression may impair pathogen clearance. If MSC exosomes primarily suppress inflammation without possessing antibacterial activity, their use during stages with high bacterial burden may be detrimental to infection control. Therefore, MSC exosomes are better suited as “immune repair tools after antimicrobial therapy, “ or used in combination with antibiotics, anti-biofilm materials, and bone repair scaffolds. The ideal strategy is not simply inhibiting NLRP3, but utilizing MSC exosomes to achieve a balance among reducing pyroptosis, promoting angiogenesis, improving osteogenesis, and modulating macrophage polarization. Therefore, the core value of MSC-related NLRP3 research lies in advancing osteomyelitis treatment from “suppressing inflammation” to “reconstructing a reparative bone immune microenvironment.” Future research needs to further clarify the optimal combination of MSC exosomes from different sources, with different engineering approaches and different miRNA cargoes, in terms of infection control, inflammation suppression, and bone repair.

### 4.7 Vascular endothelial cells

The development and progression of osteomyelitis are closely associated with local microcirculatory disturbances. Following infection, blood vessels in the medullary cavity and periosteum may exhibit endothelial activation, increased permeability, leukocyte adhesion, microthrombus formation, and tissue hypoxia. Impaired blood supply hinders antibiotic and immune cell access to infectious foci, further promoting necrotic bone formation and infection persistence (101). Although direct evidence for endothelial NLRP3 in osteomyelitis is relatively limited, from the perspective of infectious inflammation and ischemic injury mechanisms, endothelial NLRP3 likely participates in exacerbation of the osteomyelitis microenvironment (102, 103).

The main roles of NLRP3 in vascular endothelium can be summarized in three aspects. First, infectious and inflammatory stimuli induce endothelial cells to produce ROS and mitochondrial damage, thereby activating the NLRP3 inflammasome. Second, following NLRP3 activation, IL-1β and IL-18 production enhances endothelial adhesion molecule expression, promoting neutrophil and monocyte migration toward the infection site. Third, if endothelial cells undergo pyroptosis or inflammatory damage, vascular barrier integrity decreases, local exudation and microthrombus formation worsen, ultimately leading to tissue hypoxia and bone necrosis. Reviews on the interaction between NLRP3 and cell death indicate that NLRP3 activation is not only associated with pyroptosis but can also cross-talk with apoptosis, necroptosis, and other cell death modalities, forming a complex inflammatory cell death network (10, 104, 105). In osteomyelitis, the significance of endothelial NLRP3 is particularly evident in the “inflammation–ischemia–necrosis” closed loop. Infectious stimuli cause endothelial activation and inflammatory cell migration; local tissue edema and microthrombi further reduce blood supply; hypoxia induces ROS and cell death; and these ultimately generate more DAMPs, further activating NLRP3 (106). This process explains why osteomyelitis is often accompanied by necrotic bone formation, and why antibiotic therapy alone has limited efficacy in hypoperfused lesions. If antibiotics cannot adequately reach necrotic bone and biofilm regions, pathogens persist. If endothelial inflammation cannot be resolved, local ischemia and bone necrosis further expand the infectious niche.

### 4.8 T cells

Although osteomyelitis is initiated by innate immune recognition of pathogens, T cells also participate in antimicrobial defense and infection-related bone remodeling. Inflammasome-derived IL-1β promotes the recruitment, expansion, or activation of IL-17-producing T cells, particularly enhancing γδ T cell IL-17 responses, and may support Th17 cell responses in appropriate cytokine environments; IL-18 can promote IFN-γ production by antigen-specific T cells through mechanisms such as enhancing IL-12 receptor signaling (28, 107). In a mouse model of *S. aureus* surgical site infection, γδ T cells were the main source of IL-17 at the infection site, and NLRP3 deficiency or IL-1R signaling defects both attenuated local γδ T cell IL-17 responses and led to reduced bacterial clearance (28). Although this evidence does not derive directly from bone infection models, it suggests that NLRP3 downstream cytokines may participate in host defense in osteomyelitis by modulating T cell responses.

T cell responses may also promote infection-related bone destruction. IL-17 promotes neutrophil recruitment and stimulates bone matrix cells such as osteoblasts and osteocytes to increase inflammatory mediator and RANKL expression, providing favorable conditions for osteoclast differentiation and bone resorption (29). Activated T cells themselves can also express RANKL and directly promote osteoclastogenesis, linking persistent adaptive immune activation to bone loss (108). On the other hand, Th1 cell-derived IFN-γ can enhance macrophage antimicrobial effects, but sustained or excessive Th1 responses may also maintain local inflammatory stress. Regulatory T cells may limit immune-mediated tissue damage, but excessive immunosuppression may also impair bacterial clearance. In *S. aureus*-induced experimental acute hematogenous osteomyelitis, different T cell receptor Vβ subsets have been observed to exhibit distinct dynamics including early expansion, contraction, and subsequent apoptosis, while IL-2 and IFN-γ responses in perilesional tissues appeared later and lasted for shorter durations than innate inflammatory cytokines such as IL-1, IL-6, and TNF-α (109). These results suggest that *S. aureus* may reshape T cell immunity during bone infection through superantigen-like effects and dysregulated cytokine responses. Therefore, the relationship between T cells and NLRP3 is likely indirect and bidirectional: inflammasomes in myeloid cells and bone-resident cells modulate T cell recruitment, differentiation, and effector functions by altering the local cytokine environment, while T cell-derived IL-17, IFN-γ, and RANKL feed back to act on macrophages, neutrophils, osteoblast-lineage cells, and osteoclast precursors.

### 4.9 Crosstalk among different cells

The above cell types do not function in isolation. The NLRP3 inflammasome in osteomyelitis is more like a common regulator of a multicellular network, and its effects cannot be fully explained by any single-cell experiment. Macrophage experiments *in vitro* may show that *S. aureus* suppresses NLRP3 responses, while infected bone tissue assays may reveal overall elevation of NLRP3, caspase-1, and GSDMD. These findings are not contradictory, as NLRP3 signals in infected bone tissue arise from multiple cell types, stages, and spatial regions. Macrophage NLRP3 defense may be insufficient, but pyroptosis and inflammatory responses in osteoblasts, osteocytes, and osteoclast precursors may still be enhanced. NLRP3 may be insufficient to clear pathogens in early infection, but may participate in bone destruction and repair impairment in the chronic phase. Overall, NLRP3 in osteomyelitis is not a single-cell, single-stage, or single-effect pathway, but a multicellular regulatory network spanning immune cells, bone cell lineages, and the vascular system. Its pathological significance cannot be simplistically summarized as “activation exacerbates osteomyelitis, “ but should be understood as follows: when infection is not cleared, NLRP3 may be insufficient to achieve effective antimicrobial activity; under persistent infectious stimulation, NLRP3 may amplify bone destruction through IL-1β, IL-18, GSDMD-mediated pyroptosis, and RANKL-related osteoclast pathways. Therefore, future NLRP3-targeted therapy for osteomyelitis should be based on stratification by cell type, infection stage, and inflammasome activation level. Only by clarifying which cell type’s NLRP3 at which specific stage truly drives bone destruction can the transition from broad anti-inflammation to precision bone immune regulation be achieved (**Table 1**).

**Table 1:** Roles of different cell types in osteomyelitis and NLRP3-related mechanisms.

Cell type	Role in osteomyelitis	NLRP3-related mechanisms
Macrophages	Participate in early pathogen recognition and antimicrobial defense; can also amplify inflammation and bone destruction in chronic infection.	Bacterial components prime NLRP3/pro-IL-1β expression via TLR/NF-κB; subsequent K^+^ efflux, ATP, ROS, mitochondrial damage activate NLRP3, promoting caspase-1, IL-1β/IL-18 release and GSDMD pyroptosis. *S. aureus* may suppress or delay macrophage NLRP3 response, causing “insufficient defense and persistent inflammation.”
Neutrophils	Bactericidal and infection clearance in acute phase; persistent infiltration in chronic phase can damage bone matrix, endothelium, and osteoblasts.	NLRP3 primarily promotes neutrophil recruitment indirectly via IL-1β released from other cells; neutrophil IL-1β release can be NLRP3-dependent or independent (via RIPK3, serine proteases).
Osteoblasts	Bone-forming cells; also inflammatory signaling cells; impairment leads to insufficient bone repair.	*S. aureus* can invade osteoblasts; inflammatory stimuli activate NLRP3-caspase-1-GSDMD axis, inducing pyroptosis, releasing inflammatory cytokines, inhibiting osteogenic programs (RUNX2, ALP, OCN); RANKL elevation promotes osteoclastogenesis.
Osteocytes	Located in lacunae and canalicular network; sustain deep inflammation and act as danger signal sources in chronic osteomyelitis.	Hypoxia, acidification, ROS, and toxins can induce NLRP3-related pyroptosis; pyroptosis releases IL-1β, IL-18, DAMPs, sustaining deep bone matrix inflammation and potentially promoting osteoclast activation via RANKL.
Osteoclasts & Precursors	Direct executors of infectious bone resorption, leading to bone destruction and necrotic bone formation.	NLRP3 enhances RANKL/RANK signaling via IL-1β, promoting osteoclast differentiation; pyroptosis-released DAMPs, ATP, HMGB1 further activate osteoclast precursors, forming a “pyroptosis—DAMPs—osteoclast enhancement” positive feedback.
BMSCs	Responsible for bone repair and regeneration; chronic inflammation impairs proliferation, migration, and osteogenic differentiation.	Inflammatory environment activates NLRP3, increasing IL-1β/IL-18, inhibiting MSC osteogenic differentiation; MSC exosomes, miR-223, or engineered EVs can negatively regulate NLRP3, reduce pyroptosis, and promote bone repair.
Vascular Endothelial Cells	Involved in microcirculatory disturbance, inflammatory cell migration, local ischemia, and necrotic bone formation.	Infection, ROS, and mitochondrial damage activate endothelial NLRP3, promoting IL-1β/IL-18 release, adhesion molecule expression, and leukocyte migration; endothelial pyroptosis or injury leads to vascular barrier breakdown, exudation, microthrombi, and hypoxia, forming an “inflammation—ischemia—necrosis” loop.
T cells	Coordinate adaptive antimicrobial responses; enhance neutrophil recruitment and osteoclastogenesis; limit tissue damage	NLRP3 downstream IL-1β and IL-18 from myeloid or bone cells may influence γδ T, Th17, and Th1 responses; T cell-derived IL-17, IFN-γ, and RANKL feed back to neutrophils, macrophages, osteoblast-lineage cells, and osteoclast precursors

## 5 Key mechanisms of nlrp3-mediated bone immune imbalance

### 5.1 IL-1β/RANKL/osteoclast axis

IL-1β is one of the most important downstream effector factors of the NLRP3 inflammasome and a core mediator linking infectious inflammation to bone destruction. Following NLRP3 inflammasome activation, caspase-1 cleaves pro-IL-1β into mature IL-1β (110). Once released into the local osteomyelitis microenvironment, mature IL-1β acts on osteoblasts, osteocytes, macrophages, synovium-like cells, and osteoclast precursors, inducing activation of NF-κB, mitogen-activated protein kinase (MAPK), and other signaling pathways, thereby enhancing RANKL expression, promoting osteoclast differentiation, and amplifying local inflammatory responses (106, 111, 112). Thus, NLRP3 does not merely initiate inflammation, but converts “infection recognition” into “enhanced bone resorption”.

The RANKL/RANK/osteoprotegerin (OPG) system is the core pathway regulating osteoclast formation. Under normal conditions, osteoblasts and osteocytes express RANKL while simultaneously secreting OPG as a decoy receptor to limit RANKL binding to RANK, thereby maintaining the balance between osteoclastogenesis and bone formation. As reviewed by Galliera, In osteomyelitis, sustained elevation of pro-inflammatory cytokines such as IL-1β, TNF-α, and IL-6 disrupts the RANKL/OPG balance, making osteoclast precursors more readily differentiate into mature osteoclasts (113). Mature osteoclasts subsequently degrade bone mineral and organic matrix through acidification of the sealing zone, release of cathepsin K, and MMP9, leading to bone resorption, cortical bone destruction, and sequestrum formation (114). That is, bone destruction in osteomyelitis does not result from bacteria directly “dissolving” bone, but rather from infectious inflammation reshaping osteoclast differentiation and functional programs. Regarding the relationship between NLRP3 and osteoclast formation, different studies present comparable evidence. On one hand, some studies suggest that the NLRP3 inflammasome promotes bone resorption under infectious or inflammatory conditions through IL-1β. Studies showing that bone matrix components act as tissue-specific DAMPs activating the NLRP3 inflammasome and promoting osteoclast differentiation demonstrate that bone matrix degradation products themselves can serve as NLRP3-activating stimuli and drive osteoclast formation (115, 116). These results indicate that, after bone tissue is infected and destroyed, bone matrix-released products are not merely pathological consequences but can also feed back as danger signals to sustain inflammation and bone resorption (117). On the other hand, some studies suggest that NLRP3 does not always act as a unidirectional promoter of RANKL-induced osteoclast differentiation. For example, studies have found that the NLRP3 inflammasome has complex effects on osteoclast formation under different stimulation conditions; in the presence of infectious stimuli such as LPS, NLRP3-related IL-1β production more readily tips bone homeostasis toward resorption, whereas under RANKL-alone induction conditions, its effects may be limited by cell state and microenvironmental factors (118, 119). These findings suggest that NLRP3 cannot be simplistically understood as an “osteoclast differentiation switch.” More accurately, NLRP3 is a regulatory node that amplifies osteoclast responses in infectious or inflammatory contexts, its effects depending on the combined presence of pathogen stimuli, NF-κB priming, IL-1β release, and bone matrix DAMPs.

*S. aureus* osteomyelitis provides a typical disease scenario for the IL-1β/RANKL/osteoclast axis. In early infection, macrophages and neutrophils are recruited to the lesion, producing IL-1β, TNF-α, IL-6, and other inflammatory cytokines. Osteoblasts and osteocytes, stimulated by these factors, upregulate RANKL expression. Osteoclast precursors show enhanced differentiation under the influence of macrophage colony-stimulating factor (M-CSF) and RANKL (37, 120). Meanwhile, bone matrix destruction caused by *S. aureus* infection releases additional DAMPs, further activating NLRP3. This establishes a positive feedback loop of “pathogen stimulation—NLRP3 activation—IL-1β release—RANKL elevation—osteoclast enhancement—bone matrix DAMP release—NLRP3 reactivation.” This mechanism explains an important phenomenon in osteomyelitis imaging and pathology: osteomyelitis lesions often show coexisting bone destruction and new bone formation, but osteoclastic resorption predominates in actively inflamed areas. Even after antibiotics have reduced pathogen burden, the local IL-1β/RANKL axis may remain active for a short period, causing continued bone destruction. Therefore, the significance of NLRP3-targeted therapy on this mechanistic axis is not to replace antimicrobial therapy, but to limit pathological osteoclast responses after infection control, reducing irreversible bone defects. However, this axis also carries therapeutic risks. In early infection, IL-1β promotes neutrophil recruitment and bacterial control; premature or excessive inhibition of NLRP3 or IL-1β may impair host antibacterial capacity (121–123). Therefore, interventions targeting the IL-1β/RANKL/osteoclast axis should be stage-dependent.

### 5.2 GSDMD/pyroptosis/DAMPs axis

Pyroptosis is a form of inflammatory programmed cell death mediated by the Gasdermin family. Unlike apoptosis, pyroptosis is highly inflammatory, and the ATP, HMGB1, mtDNA, cytosolic proteins, and other DAMPs released by dying cells continue to stimulate surrounding cells, generating inflammatory amplification effects (32, 124–126). In osteomyelitis, pyroptosis has special pathological significance. Infectious lesions simultaneously contain PAMPs and DAMPs, and macrophages, osteoblasts, osteocytes, and some endothelial cells may all be affected by the NLRP3–caspase-1–GSDMD pathway. The more pyroptotic cells there are, the richer the local DAMPs; the richer the DAMPs, the more easily NLRP3 activation is sustained; sustained NLRP3 activation further induces more pyroptosis (10, 81, 86). Relatively direct evidence supporting this mechanism exists for *S. aureus* osteomyelitis. Researchers examined infected bone fragments from osteomyelitis patients and *S. aureus*-induced mouse osteomyelitis models, finding elevated expression of pyroptosis-related proteins such as NLRP3, caspase-1, and GSDMD in infected bone tissue; further use of pyroptosis inhibitory strategies attenuated *S. aureus*-induced inflammatory responses and bone destruction (80). This study clearly proposed that pyroptosis participates in the pathological process of *S. aureus*-induced osteomyelitis. The importance of this evidence lies in advancing NLRP3 from a general inflammatory pathway to the cell death mechanism level of infectious bone destruction. Thus, pyroptosis not only increases inflammatory cytokine levels but also transforms the entire bone microenvironment into a state of “excess danger signals”.

It is worth noting that pyroptosis is not absolutely harmful. In certain intracellular infection scenarios, pyroptosis can expose intracellular niches exploited by pathogens, re-exposing them to phagocytes and antimicrobial molecules, thus having defensive significance (127). Therefore, therapies targeting GSDMD or pyroptosis similarly cannot be simplistically understood as “completely inhibiting cell death.” What truly needs to be suppressed in osteomyelitis is persistent, spreading, pathological pyroptosis that cannot effectively clear pathogens but continuously releases DAMPs. If pyroptosis is excessively inhibited in early infection, the host’s ability to restrict intracellular pathogens may be affected. If abundant pyroptosis and DAMP release persist after infection control, pyroptosis inhibition may help interrupt the inflammatory positive feedback. Therefore, the core significance of the GSDMD/pyroptosis/DAMPs axis lies in explaining why osteomyelitis can easily transition from acute infection to chronic inflammatory bone destruction. It demonstrates that cell death is not the endpoint of disease, but rather a new starting point for inflammation; bone tissue necrosis is not a passive consequence, but rather an inflammatory source continuously providing DAMPs.

### 5.3 ROS/mitochondria/autophagy axis

The NLRP3 inflammasome has an important characteristic: it is not directly activated by a single pathogen component, but is highly sensitive to cellular homeostasis disruption. Mitochondrial damage, elevated ROS, mtDNA release, ionic homeostasis disturbances, and lysosomal damage can all drive NLRP3 assembly (128). The local osteomyelitis environment has long-term hypoxia, acidification, bacterial toxins, inflammatory cytokines, and necrotic tissue, which together cause mitochondrial stress, providing NLRP3 with the metabolic basis for sustained activation. Autophagy, particularly mitophagy, is an important protective mechanism limiting excessive NLRP3 activation. Under normal conditions, autophagy clears damaged mitochondria, reduces ROS and mtDNA release, and can also degrade inflammasome components and inflammatory cytokines. Reviews have noted that autophagy negatively regulates the NLRP3 inflammasome by removing ROS-producing damaged mitochondria, eliminating endogenous DAMPs, degrading inflammasome components, and limiting cytokine release (129, 130). Other reviews have emphasized that autophagy inhibits the NLRP3 inflammasome by reducing ROS and clearing damaged mitochondria, serving as an important homeostatic mechanism in inflammatory metabolic diseases (131, 132). In osteomyelitis, the relationship between autophagy and NLRP3 is more complex. On one hand, moderate autophagy helps clear intracellular bacteria and damaged mitochondria, thus having both antimicrobial and anti-inflammatory effects (133, 134). On the other hand, as reviewed by Lv, persistent infection, excessive ROS, and nutritional stress may cause autophagy flux impairment, preventing cells from effectively clearing damaged mitochondria (131). Following autophagy flux impairment, ROS and mtDNA further accumulate, and NLRP3 is persistently activated. NLRP3 activation leads to pyroptosis and inflammatory cytokine release, which further damages mitochondria and the autophagy system.

### 5.4 Osteoblast inhibition axis

The NLRP3-mediated osteoblast inhibition axis mainly operates at three levels. First, NLRP3 activation produces IL-1β and IL-18, placing osteoblasts in a prolonged pro-inflammatory environment that suppresses the expression of osteogenic differentiation-related genes including RUNX2, ALP, type I collagen α1 (COL1A1), and OCN (135). Second, the NLRP3–caspase-1–GSDMD pathway can induce osteoblast pyroptosis, directly reducing the number of bone-forming cells. Third, inflammasome activation exacerbates ROS and mitochondrial damage, impairing the energy metabolism of osteoblasts and MSCs, further reducing mineralization and repair capacity. Early studies support that osteoblasts express NLRP3 and participate in bacteria-induced cell death. Researchers found that osteoblasts express NLRP3, a cytosolic PRR, and proposed that bacteria-induced osteoblast death may be an important cause of bone loss in osteomyelitis (136). Evidence for NLRP3 inhibition of osteogenesis also comes from bone defect and bone metabolic disease studies. INF39, as an NLRP3 inhibitor, was reported to promote osteoblast differentiation by inhibiting NLRP3 and reducing IL-1β production, while also affecting osteoclast formation (116). In a diabetic rat alveolar bone defect model, inhibiting the NLRP3 inflammasome reduced NLRP3, ASC, caspase-1, and IL-1β expression, and increased osteogenic markers such as RUNX2 and OCN, improving bone defect healing (137). Although these studies are not all based on infectious osteomyelitis models, they demonstrate from the osteogenic repair perspective that excessive NLRP3 activation is mechanistically linked to impaired bone formation. From a therapeutic perspective, the osteoblast inhibition axis suggests that the value of NLRP3 intervention in osteomyelitis should not be evaluated solely by anti-inflammatory effects, but should also assess bone repair endpoints, including bone volume fraction, trabecular structure, cortical bone continuity, osteogenic markers, mechanical strength, and bone defect healing quality. If an NLRP3 inhibition strategy only reduces IL-1β but fails to restore osteogenic differentiation and mineralization capacity, its clinical significance is limited. Conversely, if local drug delivery, exosomes, or bone repair scaffolds can simultaneously reduce NLRP3-mediated pyroptosis, restore MSC osteogenic potential, and limit osteoclastic resorption, they would better meet the comprehensive treatment needs of chronic osteomyelitis.

### 5.5 Mutual amplification among the four mechanistic axes

**Figure 3:**
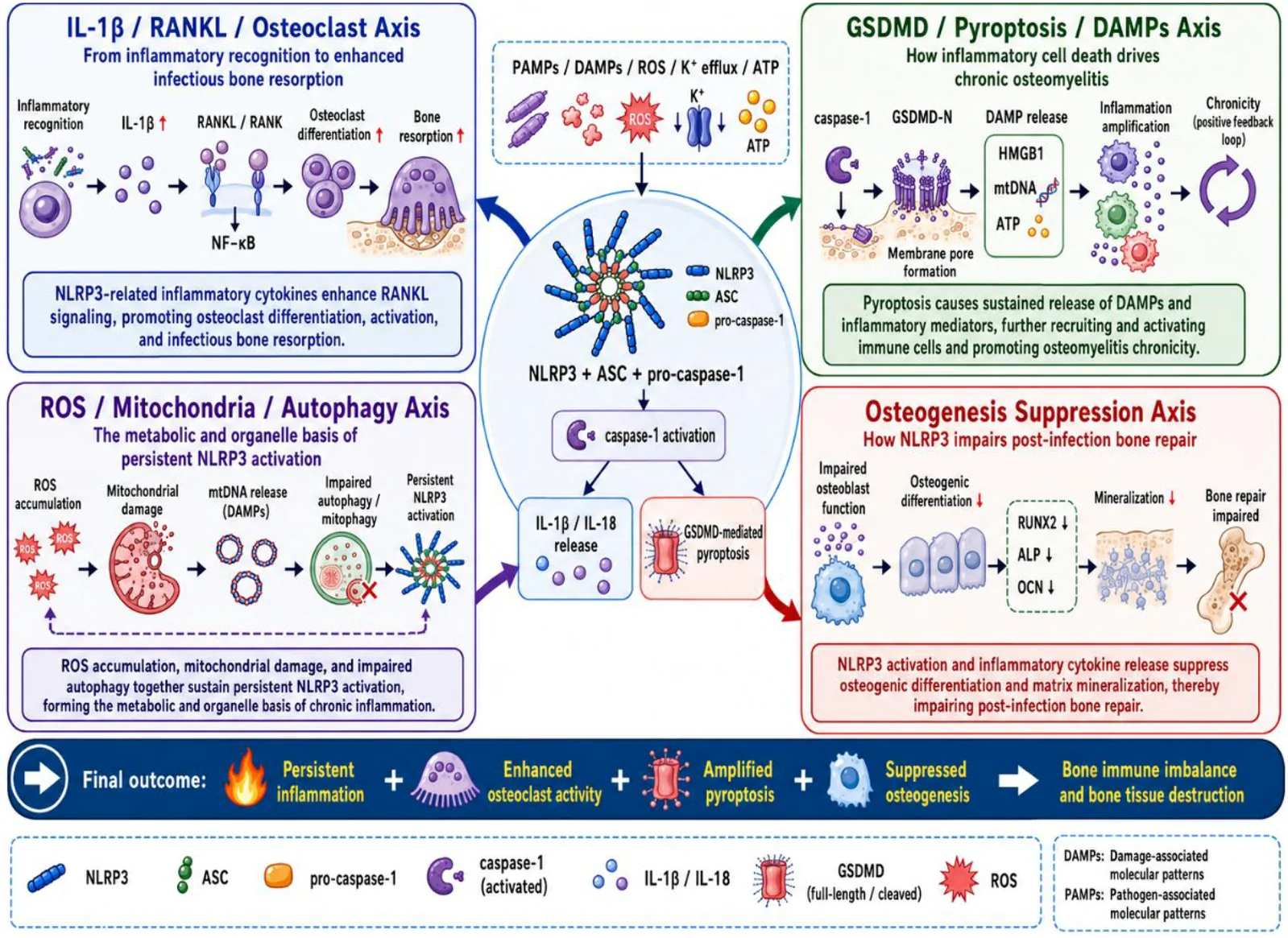
Key mechanisms of NLRP3-mediated osteoimmune imbalance in osteomyelitis. Four interconnected mechanistic axes drive the pathological network of osteomyelitis (1): IL-1β/RANKL/osteoclast axis: NLRP3-derived IL-1β upregulates RANKL expression in osteoblasts and osteocytes, promoting osteoclast differentiation and infectious bone resorption (2); GSDMD/pyroptosis/DAMPs axis: GSDMD-mediated pyroptosis releases DAMPs, which further activate NLRP3 and sustain a self-amplifying inflammatory loop (3); ROS/mitochondria/autophagy axis: mitochondrial damage and ROS accumulation drive NLRP3 activation, while impaired autophagy fails to clear damaged mitochondria, perpetuating the inflammatory stimulus; and (4) osteoblast inhibition axis: NLRP3 activation suppresses osteogenic differentiation and induces pyroptosis of osteoblasts and MSCs, leading to insufficient bone repair. These axes mutually amplify each other, forming a vicious cycle that maintains chronic inflammation, enhances bone destruction, and impairs regeneration, ultimately converting an acute infectious event into persistent bone immune imbalance.

Although the IL-1β/RANKL/osteoclast axis, GSDMD/pyroptosis/DAMPs axis, ROS/mitochondria/autophagy axis, and osteoblast inhibition axis can be discussed separately, in real osteomyelitis they are intertwined, forming a core pathological closed loop of chronic osteomyelitis: pathogens and necrotic tissue provide sustained stimuli, mitochondrial damage and autophagy impairment maintain NLRP3 activation, pyroptosis releases DAMPs amplifying inflammation, IL-1β promotes RANKL expression and osteoclast enhancement, and impaired osteoblast and MSC function leads to insufficient repair. Thus, osteomyelitis evolves from an infection event to a persistent state of bone immune imbalance. Furthermore, these mechanistic axes show distinct stage-dependence. The acute phase is mainly characterized by pathogen recognition, macrophage and neutrophil recruitment, and rapid IL-1β release. In the subacute phase, pyroptosis, DAMP release, and osteoclast activation gradually increase. The chronic phase is characterized by necrotic bone, biofilms, low-grade persistent inflammation, autophagy imbalance, and osteoblast inhibition. Therefore, the same NLRP3 pathway may have different significance at different stages. Early NLRP3 may participate in anti-infection mobilization, while late NLRP3 is more likely involved in chronic inflammation and bone destruction. Without stage distinction, therapeutic strategies are prone to errors. Finally, these mechanistic axes exhibit spatial heterogeneity. The bone marrow abscess area may be dominated by neutrophil and macrophage inflammation. The necrotic bone margin may be dominated by DAMP release, osteoclast activation, and pyroptosis. The deep bone matrix may be dominated by osteocyte damage and repair impairment. Hypoperfused areas may be dominated by hypoxia, mitochondrial damage, and poor drug accessibility. Therefore, overall detection of NLRP3 elevation in infected bone tissue does not indicate which cell type and which mechanistic axis predominates. Future studies should combine spatial transcriptomics, single-cell sequencing, multiplex immunofluorescence, and tissue regional stratification to clarify the functional differences of NLRP3 at different spatial sites. Only by placing NLRP3 in the bone immune network for stage- and mechanism-based regulation can inflammasome-targeted therapy truly become a feasible path for precision treatment of chronic osteomyelitis (**Figure 3**).

## 6 Drug development and intervention strategies targeting NLRP3 as a therapeutic target

### 6.1 Direct NLRP3 small-molecule inhibitors

Direct inhibition of NLRP3 is the most target-logical strategy. As the inflammasome sensor, NLRP3 is upstream of caspase-1, IL-1β, IL-18, and GSDMD-mediated pyroptosis. If NLRP3 assembly can be selectively blocked, it should theoretically reduce both IL-1β/IL-18 release and pyroptosis simultaneously. MCC950 is currently the most extensively studied NLRP3 small-molecule inhibitor and an important starting point for understanding NLRP3 drug development. MCC950 was reported to selectively inhibit the NLRP3 inflammasome at nanomolar concentrations, blocking canonical and non-canonical NLRP3 activation without significantly affecting AIM2, NLRC4, or NLRP1 inflammasomes (138). Further mechanistic studies showed that MCC950 directly acts on the ATP hydrolysis-related region of the NLRP3 NACHT domain, thereby inhibiting ATP hydrolysis, NLRP3 oligomerization, and inflammasome assembly. Drug mechanism reviews have also noted that MCC950 and its analogs mainly bind to the NLRP3 NACHT domain and interfere with ATP binding or ATP hydrolysis to promote NLRP3 auto-inhibition (139–141). Given the presence of biofilms, necrotic bone, and hypoperfusion in osteomyelitis lesions, systemic administration may not achieve effective concentrations at the lesion and carries the risk of impairing systemic anti-infective immunity. Therefore, compared to long-term systemic administration, loading MCC950 or its modified molecules into antibiotic-loaded bone cement, biodegradable scaffolds, nanoparticles, or hydrogels for local release is more consistent with the characteristics of osteomyelitis treatment. However, MCC950 is not yet a drug maturely developed for clinical osteomyelitis treatment. First, in infectious osteomyelitis, NLRP3 may participate in both tissue damage and early antimicrobial immunity; premature or excessive inhibition carries theoretical risks. Second, *S. aureus* can attenuate NLRP3 responses in macrophages, and NLRP3 deficiency did not significantly alter bacterial load or bone homeostasis in some models (68). This indicates that direct NLRP3 inhibitors are not suitable as standalone anti-infective agents, but should be positioned as anti-inflammasome and bone-protective drugs after infection control. Third, the pharmacokinetics, safety, and clinical development pathways of MCC950 still have limitations, and subsequent drug development is improving solubility, stability, tissue distribution, and safety windows through structural optimization.

Beyond MCC950, dapansutrile, also known as OLT1177, is an orally available selective NLRP3 inhibitor that has attracted attention. Studies have shown that dapansutrile inhibits NLRP3-dependent IL-1β production and has been explored in various inflammatory disease models and clinical studies (142, 143). Although these studies are not directly targeting osteomyelitis, they demonstrate that NLRP3 small-molecule inhibitors have progressed from experimental tools to clinical translation stages. For osteomyelitis, the potential value of oral drugs like dapansutrile lies in systemically modulating inflammasome responses, particularly in patients with chronic non-bacterial osteomyelitis where the IL-1/NLRP3 axis is active. However, in active bacterial osteomyelitis, oral systemic NLRP3 inhibitors should be used cautiously and only under the premise of pathogen control and infectious focus management.

### 6.2 Caspase-1 inhibitors

Caspase-1 is the key executive enzyme downstream of the NLRP3 inflammasome. Following NLRP3 inflammasome assembly, pro-caspase-1 is recruited and undergoes autocleavage; activated caspase-1 processes pro-IL-1β and pro-IL-18, while also cleaving GSDMD to induce pyroptosis. Therefore, inhibiting caspase-1 can block the common downstream of multiple canonical inflammasomes including NLRP3, AIM2, and NLRC4. For osteomyelitis, a disease potentially involving multiple inflammasomes and cell death pathways, caspase-1 inhibition theoretically covers a broader scope than NLRP3 inhibition alone. Pyroptosis-related studies in *S. aureus* osteomyelitis provide direct evidence for caspase-1 inhibition. In infected bone fragments and mouse osteomyelitis models, NLRP3, caspase-1, and GSDMD were elevated, and inhibition of pyroptosis-related proteins attenuated inflammatory responses and bone destruction (80). This suggests that in some *S. aureus* osteomyelitis scenarios, caspase-1/GSDMD-mediated pyroptosis does participate in tissue damage. Targeting caspase-1 could simultaneously reduce IL-1β maturation, IL-18 release, and GSDMD cleavage, theoretically helping to interrupt the pyroptosis–DAMPs–inflammatory amplification cycle. However, caspase-1 as a therapeutic target also has significant limitations. On one hand, caspase-1 is not only involved in pathological inflammation but also in early immune defense against infection. Inhibiting caspase-1 may reduce neutrophil recruitment and antimicrobial inflammation, increasing the risk of pathogen persistence. On the other hand, IL-1β maturation is not entirely dependent on caspase-1. In autoinflammatory osteomyelitis models, IL-1β can be produced through inflammasome-independent pathways (144); NLRP3 and caspase-8 may also exhibit redundancy in IL-1β processing (145–147). This means that even if caspase-1 is inhibited, caspase-8, neutrophil proteases, or other proteases may still maintain IL-1β production and bone inflammation. Thus, caspase-1 is an attractive downstream target, but its clinical translation requires precise identification of inflammasome pathway sources.

### 6.3 GSDMD and pyroptosis inhibition

Compared to NLRP3 and caspase-1, GSDMD is located at the pyroptosis execution stage. Following cleavage, GSDMD forms membrane pores, leading to cellular content release, IL-1β and IL-18 efflux, and pyroptosis. Pyroptosis in osteomyelitis not only causes cell death but also amplifies inflammation and promotes osteoclast activation through DAMP release. Therefore, GSDMD or pyroptosis inhibition strategies are closer to tissue protection endpoints, particularly suitable for reducing post-infection inflammatory bone destruction. In the *S. aureus* osteomyelitis study, pyroptosis inhibition alleviated bone destruction, providing direct evidence for anti-pyroptotic therapy (80). This study suggested that GSDMD-related pyroptosis in infected bone tissue is not merely a concomitant phenomenon but may participate in bone injury formation. If GSDMD-mediated pore formation can be inhibited, it should theoretically reduce cell lysis, DAMP release, and inflammatory cascade amplification, thereby improving the bone tissue microenvironment.

Regarding drugs, disulfiram is an already approved medication for alcohol dependence that has recently been found to inhibit GSDMD pore formation. Studies have shown that disulfiram covalently modifies key cysteine residues of GSDMD, blocking GSDMD-mediated pore formation and inhibiting pyroptosis and inflammatory cytokine release. Although this evidence mainly comes from sepsis, acute inflammation, or other organ injury models rather than osteomyelitis, it provides a pharmacological basis for GSDMD as a drug target (148). GSDMD targeting has a potential advantage in osteomyelitis: it may be closer to “reducing tissue damage” without completely shutting down all upstream pathogen recognition signals. Theoretically, NLRP3, AIM2, or other inflammasomes could still be activated and produce some immune signals, but GSDMD pore formation and pyroptotic cell lysis would be restricted, reducing DAMP release and inflammatory spread (149, 150). However, this advantage still requires experimental validation because GSDMD pores are not only involved in pyroptosis but also in IL-1β release; blocking GSDMD may simultaneously reduce protective inflammatory cytokine release, affecting early infection defense (151). Therefore, anti-pyroptotic therapy is better positioned for chronic osteomyelitis or the residual inflammatory phase after debridement, rather than the acute infection phase before pathogen control.

### 6.4 IL-1β/IL-1R blockade

IL-1β is one of the most important downstream effector factors of the NLRP3 inflammasome and a key mediator linking inflammation to bone destruction. IL-1β promotes neutrophil recruitment, enhances RANKL expression, drives osteoclast differentiation, and suppresses osteogenic repair. Since IL-1R antagonist anakinra, anti-IL-1β monoclonal antibody canakinumab, and IL-1 trap rilonacept are already clinically available, IL-1 axis blockade has a more realistic translational foundation than direct NLRP3 inhibition (152). Autoinflammatory bone diseases provide important clinical evidence for IL-1 blockade. Chronic non-bacterial osteomyelitis (CNO) and chronic recurrent multifocal osteomyelitis (CRMO) are autoinflammatory bone diseases whose pathogenesis is closely related to innate immune abnormalities, imbalanced IL-1β release, and dysregulated inflammatory cytokine networks (153, 154). Reviews have noted that CNO/CRMO involves TLR4/MAPK/inflammasome signaling and pro-inflammatory/anti-inflammatory cytokine imbalances, and some refractory patients may be considered for IL-1-targeted therapies such as anakinra or canakinumab (155–157). Deficiency of IL-1 receptor antagonist (DIRA) is a more typical IL-1-driven bone inflammatory disease, presenting as early-onset multifocal sterile osteomyelitis, periostitis, and skin inflammation; case reports and systematic reviews have shown that recombinant IL-1 receptor antagonist anakinra can bring significant clinical improvement (158, 159). These evidences indicate that the IL-1 axis has clear therapeutic value in sterile or autoinflammatory osteomyelitis. However, infectious osteomyelitis differs from CNO/CRMO in that live pathogens are present. IL-1β has dual roles in *S. aureus* osteomyelitis: on one hand, IL-1R/MyD88 signaling helps locally control bacterial replication; on the other hand, the same pathway promotes osteoclast-mediated bone damage (160). Therefore, IL-1 blockade in infectious osteomyelitis cannot simply replicate the experience from autoinflammatory bone diseases. If IL-1 blockade is used when pathogen burden is high, abscesses are undrained, or sequestra are not removed, it may impair the host’s ability to control infection.

### 6.5 ROS, mitochondrial, and autophagy modulation

NLRP3 is highly sensitive to mitochondrial damage, increased ROS, mtDNA release, and autophagy impairment. The local osteomyelitis environment has long-term hypoxia, acidification, toxins, inflammatory cytokines, and necrotic tissue, making mitochondrial damage an important basis for sustained NLRP3 activation. Therefore, modulating ROS, protecting mitochondria, and restoring autophagy flux represent an important class of strategies for indirectly inhibiting NLRP3. There is a bidirectional regulatory relationship between autophagy and NLRP3. Autophagy clears damaged mitochondria, reducing ROS and mtDNA release, thereby inhibiting NLRP3; simultaneously, autophagy can also degrade inflammasome components and inflammatory cytokines, limiting inflammatory response amplification. Reviews have noted that autophagy negatively regulates the NLRP3 inflammasome by removing ROS-producing damaged mitochondria, clearing DAMPs, and modulating inflammasome components (129, 131). This mechanism is particularly important for osteomyelitis, because mitochondrial damage and oxidative stress can persist locally even when pathogen burden decreases, and DAMPs and ROS can maintain low-grade inflammasome activation. The advantage of ROS/mitochondria/autophagy modulation lies in its relatively upstream and milder effects, potentially improving the function of osteoblasts, osteocytes, and MSCs simultaneously while reducing excessive NLRP3 activation. Its disadvantage is the lack of target specificity and complex network of effects, making it difficult to precisely predict anti-infective impacts (161). Therefore, this type of strategy is better suited for combination with antibiotics, debridement, and local bone repair materials rather than as a standalone therapy.

### 6.6 MSC-derived exosomes and miRNA therapy

Bone marrow mesenchymal stem cells and their exosomes offer another approach to NLRP3 therapy. Unlike small-molecule inhibitors, MSC-derived exosomes can not only carry miRNAs, proteins, and lipids to modulate inflammation but also promote macrophage polarization, angiogenesis, osteogenic differentiation, and tissue repair (162). For osteomyelitis, this multi-target effect is attractive because treatment requires not only reducing inflammation but also reconstructing the bone repair microenvironment. Among NLRP3-related miRNAs, miR-223 has received considerable attention. Studies in various inflammatory diseases suggest that miR-223 can negatively regulate NLRP3 expression and limit inflammasome activation. Research demonstrating that MSCs or MSC-derived exosomes attenuate inflammation and tissue damage through the miR-223/NLRP3 axis provides a mechanism that can be leveraged for osteomyelitis (99). Although this study was not conducted in an infectious osteomyelitis model, it demonstrates that MSC-related miRNAs can regulate the NLRP3 inflammasome, providing a theoretical basis for using exosomes to deliver NLRP3-negative-regulating molecules in osteomyelitis. Evidence more directly related to osteomyelitis comes from studies on FTO-engineered MSC-derived EVs. This study showed that FTO-EVs inhibited *S. aureus*-related stimuli-induced autophagy and NLRP3-mediated pyroptosis in osteoblasts and osteocytes, and alleviated bone injury in a mouse osteomyelitis model (86). This result suggests that exosomes can not only serve as anti-inflammatory carriers but also promote repair by protecting osteoblasts and osteocytes, reducing pyroptosis, and improving the bone matrix environment. The advantage of MSC exosome therapy lies in its pleiotropic effects, but this is also its complexity. If exosomes primarily suppress inflammation without antibacterial activity, their use before pathogen control may reduce host clearance capacity. Therefore, MSC exosomes are better suited as reparative immunomodulatory tools after infection control, or in combination with antibiotics and anti-biofilm materials. Future designs could incorporate “antibacterial + anti-pyroptotic + osteogenic-promoting” composite exosomes or biomimetic nanovesicles. However, clinical translation of MSC exosomes still faces challenges including standardization of preparation, drug loading efficiency, *in vivo* distribution, immunological safety, and long-term efficacy.

### 6.7 Local drug delivery systems

One of the key difficulties in pharmacological treatment of osteomyelitis is poor local drug accessibility at the lesion. Necrotic bone lacks blood supply, biofilms reduce antibiotic penetration, and abscess cavities and hypoperfused areas make it difficult for systemic drug concentrations to stably reach effective levels. If NLRP3 inhibitors are administered systemically, they may cause systemic immunosuppression. Therefore, local drug delivery systems may represent the most practically translational pathway for NLRP3-targeted therapy in osteomyelitis. Traditional local treatment for osteomyelitis often uses antibiotic-loaded bone cement or biodegradable carriers releasing vancomycin, gentamicin, and other antimicrobial agents. In the future, composite drug delivery systems can be designed: early release of high-concentration antibiotics to rapidly reduce pathogen burden; middle- and late-stage release of NLRP3 inhibitors, GSDMD inhibitors, antioxidants, or osteogenic-promoting molecules to reduce pyroptosis, inhibit pathological osteoclastogenesis, and promote bone repair. Such a temporal sequencing design aligns with the logical course of osteomyelitis: first control infection, then modulate inflammation and repair bone defects. Hydrogels, nanoparticles, biodegradable scaffolds, hydroxyapatite composite materials, and 3D-printed bone repair materials can all serve as local delivery carriers for NLRP3-related drugs (163–165). The infected bone microenvironment features acidity, elevated ROS, abundant proteases, and active inflammatory cytokines, which can be exploited to design stimuli-responsive materials (166). For example, ROS-responsive nanocarriers can release anti-inflammasome molecules in infection areas with high oxidative stress; pH-responsive hydrogels can release antibiotics and NLRP3 inhibitors in acidic abscess cavities; bone-targeting bisphosphonate-modified nanoparticles can enhance drug enrichment on bone surfaces (167).

Local delivery can also reduce the systemic risks of NLRP3 inhibitors. Since NLRP3 participates in systemic innate immune responses, long-term systemic inhibition may increase infection risk; local delivery can achieve higher drug concentrations in the lesion while reducing exposure of the peripheral immune system (110). For infectious osteomyelitis, this is particularly important. The ideal local NLRP3 therapy should not completely block immune responses in early infection, but after antimicrobial agents reduce pathogen burden, continuously and slowly release anti-pyroptotic and pro-repair factors to reduce inflammatory bone destruction (168, 169). Of course, local delivery also has challenges. First, there may be conflicts in release profiles between NLRP3 inhibitors, antibiotics, and osteogenic-promoting factors. Antibiotics typically require early high-concentration release, whereas NLRP3 inhibitors are better suited for sustained low-dose release in the middle and late stages. Second, materials themselves may cause foreign body reactions or affect osseointegration (20). Third, if biofilms and sequestra are not adequately removed, local anti-inflammation may mask active infection and increase recurrence risk. Therefore, local delivery must be based on adequate debridement and pathogen control, rather than replacing surgical management.

### 6.8 Combination therapy strategies

Treatment of osteomyelitis cannot rely on a single target. Even if NLRP3 is an important mechanistic node, simply inhibiting NLRP3 cannot clear pathogens, remove sequestra, eliminate biofilms, or reconstruct bone defects. Therefore, NLRP3-related therapy should be embedded within a combination therapy framework. The first type of combination is antibiotics combined with NLRP3 inhibitors. The logic is that antibiotics reduce pathogen burden while NLRP3 inhibitors reduce inflammatory bone destruction after infection control. If animal experiments show that combination therapy reduces GSDMD-N, IL-1β, RANKL, and bone destruction more than antibiotics alone, without increasing bacterial burden, this would support the strategy. Importantly, the timing of administration is critical. If NLRP3 inhibitors are used too early, they may impair antimicrobial immunity; if used after antibiotics have taken effect, they may be safer (110). The second type is anti-biofilm therapy combined with NLRP3 modulation. One of the core issues in chronic osteomyelitis and implant-related infections is biofilm. If biofilms are not disrupted, local inflammation modulation cannot eradicate infection. Anti-biofilm strategies include material surface anti-adhesion coatings, antimicrobial peptides, bacteriophages, DNase, biofilm matrix-degrading enzymes, and locally high-concentration antibiotics (20, 170). NLRP3 modulation can serve as a tissue protection strategy after biofilm removal, rather than replacing anti-biofilm therapy (171). The third type is anti-pyroptosis combined with osteogenic promotion. Pyroptosis inhibition can reduce DAMP release and inflammatory damage, but bone defect repair also requires osteoblasts, MSCs, angiogenesis, and bone matrix reconstruction. Therefore, GSDMD inhibitors, NLRP3 inhibitors, or IL-1 blocking agents can be combined with bone morphogenetic proteins (BMPs), vascular endothelial growth factor (VEGF), MSC-derived exosomes, hydroxyapatite scaffolds, or bioactive glass to form a dual strategy of “reducing inflammatory cell death + promoting bone regeneration” (172). The fourth type is host immune reprogramming. Chronic osteomyelitis often involves macrophage functional imbalance, with insufficient bacterial killing on one hand and sustained pro-inflammatory damage on the other. NLRP3 modulation can be combined with macrophage polarization regulation, metabolic reprogramming, mitochondrial protection, and autophagy enhancement to shift macrophages from a sustained pro-inflammatory and inefficient bactericidal state toward an effective clearance and repair-supportive state. The key evaluation endpoints for combination therapy should not be merely decreases in inflammatory cytokines. At least five categories of indicators should be observed simultaneously: bacterial burden and recurrence rate; inflammasome markers such as NLRP3/caspase-1/GSDMD/IL-1β; osteoclast markers such as TRAP, RANKL/OPG, and cathepsin K; osteogenic markers such as RUNX2, ALP, OCN, and COL1A1; and bone repair quality reflected by micro-computed tomography (micro-CT), histology, and mechanical strength. Only when “infection not worsened, inflammation decreased, bone destruction reduced, and osteogenic repair improved” are simultaneously satisfied does NLRP3 therapy have true translational value (**Table 2**).

**Table 2:** Advantages, limitations, and suitable scenarios of different intervention strategies targeting NLRP3 in osteomyelitis.

Intervention strategy	Advantages	Limitations	Suitable scenarios
Direct NLRP3 Inhibitors	Targets upstream sensor; simultaneously reduces IL-1β, IL-18 release and GSDMD pyroptosis; strong mechanistic specificity.	May impair necessary antibacterial immunity if used early; poor systemic bioavailability in hypoperfused osteomyelitis lesions.	Better suited for post-infection-control, chronic inflammation, and ongoing bone destruction stages; ideally combined with local delivery and antibiotics.
Caspase-1 Inhibitors	Blocks common downstream of multiple canonical inflammasomes; reduces IL-1β/IL-18 maturation and GSDMD cleavage.	Broader but less specific; IL-1β can be produced via alternative pathways (caspase-8, neutrophil proteases).	Suitable for osteomyelitis with clear evidence of caspase-1 activation/pyroptosis, but avoid early use when pathogen load is high.
GSDMD/Pyroptosis Inhibition	Directly blocks pyroptosis execution; reduces DAMP release and inflammation spread; closer to tissue protection endpoint.	May affect IL-1β release and early anti-infective responses; direct evidence in osteomyelitis still needed.	Suitable for chronic osteomyelitis, residual inflammation post-debridement, stages where pyroptosis and bone destruction persist despite infection control.
IL-1β/IL-1R Blockade	Existing clinical drugs, high translational feasibility; inhibits IL-1β/RANKL/osteoclast axis.	May impair IL-1β-mediated bacterial control in infectious osteomyelitis.	Better for chronic non-bacterial, autoinflammatory bone diseases; in infectious osteomyelitis, use cautiously as adjunct post-pathogen control.
ROS/Mitochondria/Autophagy Modulation	Indirectly limits persistent NLRP3 activation by improving organelle homeostasis; protects osteoblasts, osteocytes, MSCs.	Targets lack specificity; complex action network; anti-infective effects hard to predict.	Suitable for chronic lesions with prominent hypoxia, ROS elevation, autophagy impairment; combine with antibiotics and bone repair materials.
MSC Exosomes/miRNA Therapy	Simultaneously modulates inflammation, pyroptosis, macrophage polarization, angiogenesis, and osteogenic repair.	Challenges in standardization, loading efficiency, biodistribution, immune safety; single use lacks antibacterial activity.	Better as post-infection-control immune repair tool; combine with antibiotics, anti-biofilm materials, bone repair scaffolds.
Local Drug Delivery Systems	Enhances local drug concentration, reduces systemic immunosuppression risk; suits hypoperfused, necrotic bone environment.	Complex release profile design; potential foreign body reactions; cannot replace debridement and pathogen control.	Most translatable potential; suitable for sequential release of antibiotics, NLRP3 inhibitors, anti-pyroptotic agents, and osteogenic factors.
Combination Therapy	Integrates antibacterial, anti-biofilm, anti-pyroptosis, and osteogenic promotion; aligns with complex osteomyelitis pathology.	Strategy complexity requires clear dosing timing and efficacy endpoints.	Suitable for chronic, recurrent, implant-associated, or large bone defect osteomyelitis; future mainstay.

## 7 Discussion and perspectives

In summary, the NLRP3 inflammasome-mediated bone immune imbalance provides an important mechanistic perspective for understanding the chronic persistence, bone destruction, and repair impairment in osteomyelitis. Traditional osteomyelitis research has largely attributed disease core to pathogen invasion, poor antibiotic penetration into infectious foci, and the formation of sequestra and biofilms. However, the present review indicates that osteomyelitis is not simply a local infection event, but a complex bone immune disease involving pathogens, immune cells, bone cell lineages, cell death modalities, bone remodeling systems, and local microcirculation. The NLRP3 inflammasome lies at a critical intersection of this network: it can sense PAMPs and DAMPs, and through caspase-1 activation, maturation and release of IL-1β and IL-18, and GSDMD-mediated pyroptosis, it influences local anti-infective responses, inflammatory amplification, osteoclast activation, and osteogenic repair processes. Therefore, research centered on NLRP3 helps advance the understanding of osteomyelitis from “pathogen infection of bone tissue” to “infection-driven disruption of bone immune homeostasis”.

Currently, the role of NLRP3 in osteomyelitis cannot be simplistically categorized as “harmful” or “beneficial.” On one hand, studies have shown that NLRP3 can indeed be an important amplifier of osteomyelitis chronicity and bone destruction. On the other hand, NLRP3 is not the sole dominant factor in all osteomyelitis pathological processes. *S. aureus* possesses complex immune evasion capabilities, weakening host defense through intracellular survival, biofilm formation, virulence factor regulation, and metabolic reprogramming. In some models, *S. aureus* infection of macrophages does not trigger a rapid and robust NLRP3 response, and NLRP3 deficiency does not necessarily significantly alter bacterial burden or bone homeostasis. This indicates that the role of NLRP3 in osteomyelitis depends on pathogen type, infection stage, cell type, tissue spatial location, and host immune status. Ignoring these conditions and simplistically proposing “inhibiting NLRP3 to treat osteomyelitis” risks oversimplifying mechanistic judgments. Therefore, future research should regard NLRP3 as a context-dependent bone immune regulatory node rather than a unidirectional pathogenic switch. In early infection, NLRP3 and its downstream IL-1β may participate in neutrophil recruitment and pathogen restriction. After infection persists, sequestra form, and biofilms stabilize, NLRP3-related pyroptosis, DAMP release, and the IL-1β/RANKL axis are more likely to drive bone destruction. In the repair phase, sustained NLRP3 activation may suppress osteoblast and MSC function, delaying bone defect healing. Thus, determining whether NLRP3 should be intervened upon requires first clarifying the disease stage and inflammasome activation status. However, judgments regarding stage-dependent roles of NLRP3 should remain cautious, as direct *in vivo* evidence demonstrating that NLRP3 is protective in early osteomyelitis and pathogenic in chronic infection stages is still lacking. Available evidence mainly comes from conventional global knockout models, pharmacological inhibition experiments of pyroptosis, and temporal analyses of infected cells. Recent studies have shown that after *S. aureus* infection of bone marrow-derived macrophages, NLRP3 activation is relatively weak and significantly delayed, becoming more evident mainly after 18 hours of infection. NLRP3 deficiency could to some extent impair the ability of macrophages to reduce intracellular bacterial burden, but did not significantly alter bacterial burden or bone homeostasis in experimental osteomyelitis (33). In contrast, another mouse study showed that pharmacological inhibition of pyroptosis attenuated inflammatory bone damage (80). Taken together, these results are more supportive of a context-dependent working model than a definitively established temporal dichotomy: NLRP3 may participate in some antimicrobial responses in specific cells and infection contexts, but when infection is established and persistent, sustained inflammasome activation and pyroptosis may promote inflammatory bone destruction. Future studies combining inducible and cell-specific genetic models with longitudinal assessments of bacterial burden, inflammasome activity, bone resorption, and bone repair are needed to validate this model.

Although the connection between NLRP3 and osteomyelitis is becoming clearer, existing research still has notable limitations. First, there remains a gap between current experimental models and clinical chronic osteomyelitis. Many studies use acute bacterial inoculation models or *in vitro* cell stimulation models, which can effectively observe inflammatory cytokine release, pyroptosis, and osteoclast activation, but cannot fully simulate the sequestra, biofilms, hypoperfusion, implants, long-term antibiotic exposure, and recurrent relapses characteristic of clinical chronic osteomyelitis (173–178). Clinical osteomyelitis often has a long time course, marked spatial heterogeneity, and complex therapeutic interventions (179). Therefore, the pro-inflammatory effects of NLRP3 observed in acute models cannot be directly equated with its actual roles in chronic lesions. Second, current inflammasome research in osteomyelitis is heavily biased toward *S. aureus*. While *S. aureus* is indeed one of the most important pathogens in osteomyelitis and is therefore justified as the primary subject of discussion, the pathogen spectrum of osteomyelitis has significant clinical context variations. Gram-negative bacteria, polymicrobial infections, coagulase-negative staphylococci, and less common pathogens such as mycobacteria and fungi can also participate in disease pathogenesis (180). Gram-negative bacterial LPS can mediate inflammasome priming signals through TLR4, while cytosolic LPS can be directly recognized by human caspase-4/5 or mouse caspase-11 to activate the non-canonical inflammasome pathway (54). Bacterial flagellin and type III secretion system-related components mainly activate the NAIP/NLRC4 inflammasome, and cytosolic bacterial double-stranded DNA can activate the AIM2 inflammasome (181). Therefore, the inflammasome activation patterns in Gram-negative infections may differ from those in *S. aureus* infections, which are characterized mainly by TLR2-related priming, membrane-damaging toxins, and biofilms. Such differences may be more prominent in post-traumatic osteomyelitis, diabetic foot-related osteomyelitis, chronic wound-spreading infections, and immunocompromised patients. Polymicrobial osteomyelitis can simultaneously provide multiple classes of PAMPs, causing parallel or sequential activation of NLRP3, NAIP/NLRC4, AIM2, and non-canonical inflammasomes, superimposed with DAMPs from tissue necrosis, local ischemia, metabolic abnormalities, and bone matrix degradation, forming a more complex inflammatory network (181). Implant-associated infections have additional specificity: besides microbial and biofilm-related signals, wear particles, metal ions, and DAMPs from local mechanical injury and necrotic tissue may also continuously provide sterile NLRP3-activating stimuli, allowing infectious inflammation and material-related inflammation to promote each other. Studies have shown that orthopedic implant wear particles can induce NLRP3 inflammasome-dependent IL-1β release and participate in periprosthetic inflammatory osteolysis (182). It should be noted that direct studies on inflammasome responses in non-*S. aureus* osteomyelitis are still relatively limited, particularly lacking systematic comparisons between different pathogens. Therefore, it is currently inappropriate to generalize NLRP3 effects observed in one pathogen model to all types of osteomyelitis. Future studies should stratify according to pathogen species, single or mixed infection status, presence of implants, and underlying host diseases, to clarify the relative contributions of different inflammasomes to pathogen clearance, inflammatory cell death, enhanced bone resorption, and impaired bone repair.

Third, NLRP3 and IL-1β cannot be simply equated. IL-1β is a key factor in the osteomyelitis inflammatory network, but its maturation processing can be mediated by the NLRP3–caspase-1 pathway or by alternative pathways including caspase-8 and neutrophil proteases. Therefore, detecting elevated IL-1β alone does not prove that NLRP3 is the major upstream pathway. To use NLRP3 as a diagnostic marker or therapeutic target in the future, comprehensive assessment combining ASC specks, cleaved caspase-1, GSDMD-N, IL-18, and cellular localization results is necessary (183–185). Finally, most current therapeutic studies remain at the mechanistic validation or animal experiment stage, with a considerable distance from clinical translation. MCC950, dapansutrile, and other NLRP3 inhibitors, caspase-1 or GSDMD inhibition strategies, IL-1β/IL-1R blockade, and MSC-derived exosomes and local drug delivery systems all show potential value. However, infectious osteomyelitis differs from sterile inflammatory diseases, and any immunosuppressive or anti-inflammasome therapy may affect pathogen clearance. Therefore, future studies must simultaneously evaluate anti-inflammatory effects, bacterial burden, recurrence rates, and bone repair quality, rather than using only decreased inflammatory cytokines as efficacy endpoints.

Future mechanistic research on NLRP3 in osteomyelitis should shift from single-pathway validation to temporal, spatial, and cell-specific analyses. In the temporal dimension, the functional differences of NLRP3 should be examined separately during acute infection, infection persistence, chronic sequestrum/biofilm stages, and bone repair phases. Only by defining the time window can it be determined when NLRP3 inhibition is beneficial and when it may be detrimental to anti-infection. In the spatial dimension, distinctions should be made among abscess areas, sequestrum margins, periosteal reaction zones, medullary cavity inflammatory areas, deep cortical bone, and hypoperfused regions (186). Different regions have varying cellular compositions, oxidative stress levels, pathogen burdens, and drug accessibility, and NLRP3 activation levels may also differ. Future studies can combine spatial transcriptomics, multiplex immunofluorescence, single-cell sequencing, and regional tissue stratification to clarify the distribution and function of NLRP3 in different lesion spaces (187–189). At the cellular level, cell type-specific NLRP3 modulation models should be established for macrophages, osteoblasts, osteocytes, osteoclast precursors, MSCs, and vascular endothelial cells. Resolving these questions will determine whether NLRP3 can be transformed from a general inflammatory target into a precision bone immune therapeutic target.

From the perspective of diagnostic and prognostic biomarkers, clinical translation of inflammasome biology requires biomarkers capable of distinguishing active infection, inflammatory bone destruction, treatment response, and reparative bone remodeling. Currently, the diagnosis and monitoring of infectious osteomyelitis still rely on the integrated assessment of clinical presentation, C-reactive protein (CRP), erythrocyte sedimentation rate (ESR), white blood cell count, microbiological culture, histopathology, and imaging. Potential inflammasome-related markers include tissue or circulating NLRP3, ASC, active caspase-1, IL-1β, IL-18, cleaved GSDMD, extracellular ASC specks, cell-free mtDNA, and inflammasome-related EV contents. However, these direct or proximal inflammasome markers currently face common issues such as non-standardized detection methods, sensitivity to sample processing, unclear cellular origins, and insufficient disease specificity (190). Bone remodeling markers such as RANKL/OPG, TRAP5b, C-terminal telopeptide of type I collagen (CTX-I), N-terminal propeptide of type I procollagen (P1NP), and bone-specific alkaline phosphatase (BALP) may provide complementary information on bone resorption and repair status. However, their circulating levels are easily influenced by age, renal function, circadian rhythm, food intake, medications, and other bone metabolic conditions (191). Indeed, in a mouse *S. aureus* osteomyelitis model, plasma RANKL levels lacked stable correlation with imaging or histological bone destruction severity and could not reliably reflect antibiotic treatment response, suggesting that local bone tissue pathway activity may not be accurately represented by systemic circulating markers (192). Therefore, inflammasome markers and bone remodeling markers may be better suited as part of a multi-marker panel, interpreted in conjunction with microbiological, imaging, and histological findings, rather than serving as standalone diagnostic evidence. Comparing infectious osteomyelitis with chronic nonbacterial osteomyelitis (CNO) has important value. Infectious osteomyelitis is triggered by live microorganisms, involving both PAMP-driven innate immune recognition, DAMP release, biofilm formation, intracellular persistence, tissue necrosis, and local perfusion impairment. In contrast, CNO is a sterile autoinflammatory bone disease with microbiological examinations typically not supporting active infection; its main feature is dysregulated innate immunity and cytokine regulation in the absence of a clear pathogen. Some CNO cohort studies have shown that combinations of serum IL-6 and CCL11/eotaxin may help distinguish CNO from healthy controls and some differential diagnoses including bone and joint infections, but researchers have emphasized the need for validation in larger independent cohorts (193). Other studies have found elevated inflammatory proteins such as S100A8/A9, IL-18, IL-17, IL-1β, and TNF-α in CNO patients, suggesting a possible monocyte-driven autoinflammatory state; however, these markers show significant overlap across different inflammatory diseases and cannot be used alone to confirm CNO or exclude infection (194). Clinically distinguishing between them is very important, because inhibiting IL-1 or NLRP3 in CNO does not carry the same degree of pathogen clearance risk as in active infectious osteomyelitis. Therefore, for suspected bacterial osteomyelitis, inflammasome-targeted therapy should only be considered after adequate pathogen control and exclusion of uncontrolled infection.

From a therapeutic perspective, the promise of NLRP3-targeted strategies does not mean developing one drug to treat all osteomyelitis. The foundation of infectious osteomyelitis treatment remains pathogen control, including adequate antimicrobial therapy, abscess drainage, sequestrum removal, infected implant management, and anti-biofilm interventions. On this basis, NLRP3 modulation can serve an adjuvant therapeutic role (195). A more rational future treatment pathway should be staged combination intervention. In the acute infection phase, priority should be given to controlling pathogens and infectious foci, avoiding premature systemic NLRP3 inhibition that may impair necessary innate immune mobilization. After infection control, if local NLRP3/caspase-1/GSDMD activation, enhanced IL-1β/RANKL axis, and excessive osteoclast activity persist, NLRP3 inhibitors, caspase-1 inhibitors, GSDMD inhibitors, or IL-1 blockade could be considered as adjunctive therapies. During the bone defect repair phase, NLRP3 modulation should be combined with MSC-derived exosomes, osteogenic-promoting materials, angiogenesis support, and local drug delivery systems to achieve simultaneous inflammation resolution and bone regeneration. Local drug delivery systems may be the most practically translational direction for NLRP3 therapy in infectious osteomyelitis. Necrotic bone, biofilms, and hypoperfusion limit systemic drug access to lesions, while systemic NLRP3 inhibition carries immunosuppressive risks. Antibiotic-loaded bone cement, biodegradable scaffolds, ROS-responsive nanoparticles, pH-responsive hydrogels, hydroxyapatite composite materials, and bone-targeting exosome-based drug delivery can all serve as future research directions. The ideal material should have sequential release capability: early release of antimicrobial agents to reduce pathogen burden, middle release of anti-NLRP3 or anti-pyroptotic molecules to reduce DAMPs and osteoclast stimuli, and late release of osteogenic- or angiogenic-promoting factors to promote bone defect repair. Such a strategy better matches the disease course characteristics of osteomyelitis than simply systemic inflammasome inhibition.

The therapeutic concept ultimately pointed to by this review is shifting from “anti-inflammation” to “bone immune reprogramming.” Inflammation in osteomyelitis is not purely harmful; early inflammation helps limit pathogen spread. What truly requires intervention is the uncontrolled inflammasome activation, pathological pyroptosis, DAMP release, osteoclast enhancement, and osteoblast suppression that occur after infection persists. Therefore, future therapeutic goals should not be merely reducing CRP, ESR, or IL-1β, but simultaneously achieving pathogen clearance, inflammation resolution, controlled osteoclast activity, restored osteogenesis, improved vascularization, and functional reconstruction. Modulation of NLRP3 should not pursue long-term, systemic, non-selective inhibition, but rather precise intervention based on the patient’s disease stage, pathogen burden, inflammasome activation level, and bone repair status.

## 8 Conclusion

The NLRP3 inflammasome is a critical node in the osteoimmune imbalance network of osteomyelitis, with distinctly context-dependent roles: it may participate in antimicrobial defense in early infection, while in the chronic phase, it drives bone destruction and impaired repair through multiple pathways including the IL-1β/RANKL axis, GSDMD pyroptosis/DAMP release, and osteogenesis suppression. *S. aureus* immune evasion capabilities mean that NLRP3 activation does not consistently translate into effective bactericidal activity; therefore, it cannot be simply labeled as “harmful” or “beneficial.” Therapeutically, NLRP3-targeted strategies should not pursue non-specific inhibition but be embedded within a comprehensive framework based on pathogen control. Stratified intervention according to infection stage—preserving necessary immunity in the acute phase, inhibiting pathological pyroptosis and osteoclast activity in the chronic phase, and combining with osteogenic promotion in the repair phase—is more consistent with the disease logic. Local drug delivery systems, capable of precise drug release and reducing systemic immune risks, represent a highly promising translational direction. Ultimately, osteomyelitis therapy should move from “anti-inflammation” to “osteoimmune reprogramming, “ aiming to shift the infected bone microenvironment from a state of persistent damage to one of regeneration and repair. As a therapeutic target, precise NLRP3 intervention requires individualized assessment based on disease course, cell type, and inflammasome activation levels.
